# Direct evidence for ligand-enhanced activity of Cu(i) sites†

**DOI:** 10.1039/d4sc04582c

**Published:** 2024-08-16

**Authors:** Elvira Gouatieu Dongmo, Shabnam Haque, Florian Kreuter, Toshiki Wulf, Jiaye Jin, Ralf Tonner-Zech, Thomas Heine, Knut R. Asmis

**Affiliations:** a Wilhelm-Ostwald-Institut für Physikalische und Theoretische Chemie, Universität Leipzig Linnéstr. 2 04103 Leipzig Germany jiaye.jin@uni-leipzig.de ralf.tonner@uni-leipzig.de knut.asmis@uni-leipzig.de; b Institute of Resource Ecology, Research Site Leipzig, Helmholtz-Zentrum Dresden-Rossendorf Permoserstr. 15 04318 Leipzig Germany; c Faculty of Chemistry and Food Chemistry, School of Science, TU Dresden 01062 Dresden Germany thomas.heine@tu-dresden.de; d Department of Chemistry and ibs for Nanomedicine, Yonsei University Seodaemun-gu Seoul 120-749 Republic of Korea

## Abstract

Little is known about the strong mediating effect of the ligand sphere and the coordination geometry on the strength and isotopologue selectivity of hydrogen adsorption on the undercoordinated copper(i) site. Here, we explore this effect using gas-phase complexes Cu^+^(H_2_O)(H_2_)_*n*_ (with *n* ≤ 3) as model systems. Cu^+^(H_2_O) attracts dihydrogen (82 kJ mol^*−*1^) more strongly than bare Cu^+^ (64 kJ mol^*−*1^) does. Combining experimental and computational methods, we demonstrate a high isotopologue selectivity in dihydrogen binding to Cu^+^(H_2_O), which results from a large difference in the adsorption zero-point energies (2.8 kJ mol^−1^ between D_2_ and H_2_, including an anharmonic contribution of 0.4 kJ mol^−1^). We investigate its origins and the bond strengthening between Cu^+^ and H_2_ upon addition of a single H_2_O ligand. We discuss the role of the environment and the coordination geometry of the adsorption site in achieving a high selectivity and the ramifications for identifying and designing future materials for adsorptive dihydrogen isotopologue separation.

## Abstract 2

Cu(i) sites enhance their activity upon coordination with oxygen-donor ligands, as demonstrated by infrared photodissociation spectroscopy and *ab initio* calculations.

## Graphical abstract

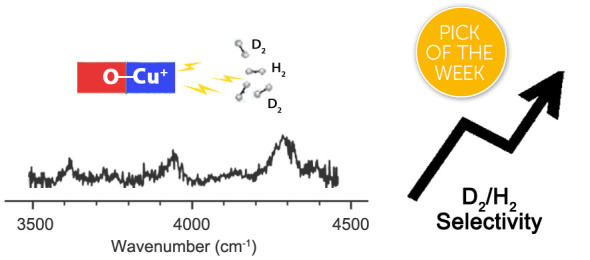

## Introduction

Porous materials containing under-coordinated Cu(i) centres with a strong tendency to adsorb dihydrogen are promising candidates for efficient H_2_/D_2_ separation.^1–5^ A benchmark material with such centres is the metal–organic framework (MOF) Cu(i)-MFU-4*l*, containing trigonal pyramidal Cu^+^ sites linked by BTDD (bis-(1*H*-1,2,3-triazolo-[4,5-*b*],[4′,5′-*i*])dibenzo-[1,4]-dioxin).^2,4,5^ Its high D_2_/H_2_ adsorption selectivity of 11 at the relatively high temperature of 100 K is due to the combination of high enthalpies of adsorption for H_2_ (31 kJ mol^*−*1^) and D_2_ (34 kJ mol^*−*1^) and the relatively high difference of these values between the two isotopologues. The latter is governed by the zero-point energy (ZPE) difference, and leads to a strong preference towards adsorption of the higher mass isotopologue.^6–8^

The rational design of high-performance separation materials requires a detailed understanding of the binding of dihydrogen isotopologues to metal centres, particularly Cu(i), which has shown a strong dihydrogen affinity in many cases.^2–4^ However, spectroscopic studies of dihydrogen coordinated to Cu(i) centres in bulk materials typically suffer from structural heterogeneity, complicating an in-depth understanding. This can be avoided by performing experiments on well-defined and isolated gas-phase complexes using vibrational action spectroscopy in combination with electronic structure calculations.^9^ This approach allows characterization of the geometric structure of relevant M–H_2_ motifs,^10^ and also provides a deeper understanding of their binding nature as well as the isotopologue selectivity.

The adsorption thermodynamics of H_2_ isotopologues at binding sites in porous materials are governed by the ZPE effects associated with the conversion of the three translational and two rotational degrees of freedom of free H_2_ into five molecule-adsorption-site vibrations.^11^ These low-frequency vibrational modes usually display strongly anharmonic behaviour and pronounced nuclear quantum effects,^12^ raising a research interest in model complexes that show the influence of these vibrational modes on dihydrogen isotopologue separation.

A recent spectroscopic and theoretical study of the Cu^+^(H_2_)_4_ complex and its isotopologues revealed a substantial red-shift of the dihydrogen stretching frequency (*ν*_HH_ = 3729 cm^*−*1^ and *ν*_DD_ = 2678 cm^*−*1^) upon complexation (*ν*_HH_ = 4162 cm^*−*1^ and *ν*_DD_ = 2994 cm^*−*1^).^13^ The exceptionally high sequential bond dissociation energy (BDE) for Cu^+^(H_2_)_*n*_ → Cu^+^(H_2_)_*n*−1_ + H_2_ with *n* = 1 of 64 kJ mol^*−*1^ reduces to only 21 kJ mol^*−*1^ with *n* = 4.^13,14^ This study motivated the investigation of H_2_-affine Cu(i) complexes with oxygen-donor ligands that can significantly increase the Cu^+^–H_2_ BDE.^8,14,15^ The aqua complex Cu^+^(H_2_O), arguably the simplest such system, represents a useful model for an undercoordinated adsorption site in a zeolite or a MOF. The experimental BDE of the Cu^+^–H_2_ bond in Cu^+^(H_2_O)(H_2_) is 82 kJ mol^*−*1^,^14^ which is 18 kJ mol^*−*1^ larger than that in Cu^+^(H_2_). Such high BDEs are desirable for adsorptive separation of dihydrogen isotopologues, both because they allow higher operating temperatures and also because they typically result in larger ZPE differences, which lead to a higher selectivity.

In this paper, we disentangle why a single H_2_O ligand already markedly strengthens the Cu(i)–H_2_ interaction. We first investigate the interaction of dihydrogen and its isotopologues with Cu^+^(H_2_O) by ion-trap mass spectrometry at variable temperature. We then combine cryogenic ion-trap vibrational action spectroscopy^16,17^ of Cu^+^(H_2_O)(H_2_)_2_ isotopologues with quantum-chemical calculations to characterize their structures and vibrational properties. Finally, we make use of bond analysis methods to rationalize the strong chemical bonding of H_2_ to the Cu(i) centre as well as the large ZPE difference of the dihydrogen isotopologues in order to identify the relevant factors that govern the isotopologue selectivity of dihydrogen adsorption.

## Results and analysis

### Ion-trap mass spectrometry

We begin our investigation by characterizing the adsorption yield of H_2_ and D_2_ on mass-selected Cu^+^(H_2_O) cations confined (on average) for 50 ms in an ion-trap reactor under multiple-collision conditions at ion-trap temperatures ranging from 295 K to 15 K. Under these conditions, Cu^+^(H_2_O)(H_2_/D_2_)_*n*_ complexes with *n* ≤ 4 can be formed *via* three-body collisions. Their relative abundances are detected using time-of-flight (TOF) mass spectrometry. Integration over the corresponding normalized TOF-MS peaks yields the relative ion yields (see Fig. S1† for all TOF mass spectra and relative ion yields).

**Fig. 1:**
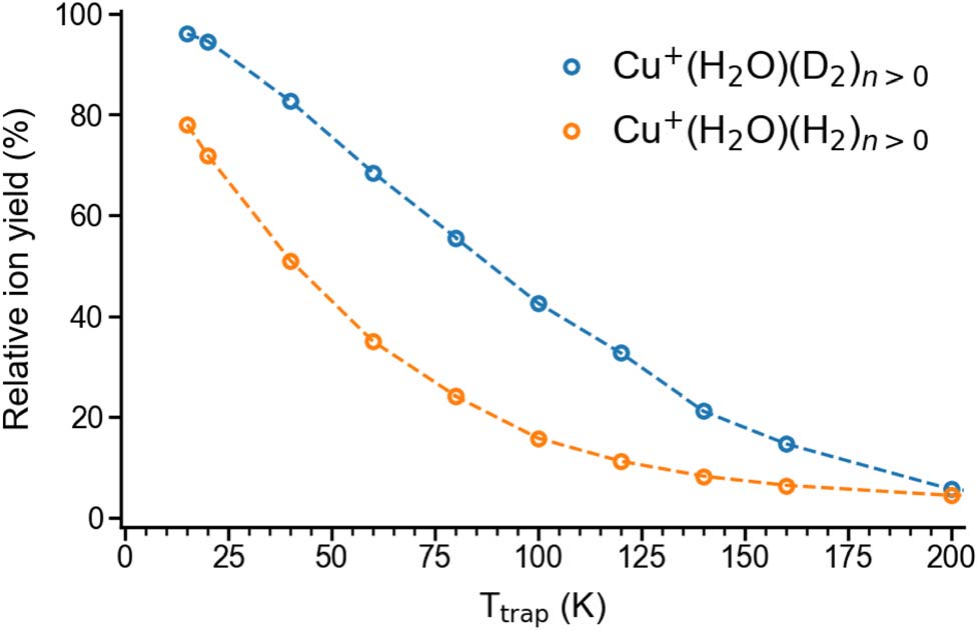
Total relative ion yield of Cu^+^(H_2_O)(H_2_)_*n*>0_ and Cu^+^(H_2_O)(D_2_)_*n*>0_ for ion trap temperatures ranging from 15 K to 200 K, obtained from the TOF mass spectra shown in Fig. S1.† The TOF mass spectra were recorded after storing Cu^+^(H_2_O) cations on average 50 ms in a temperature-controlled ion trap filled with roughly 1 mbar of H_2_ or D_2_.

Summation over all ion yields with *n* > 0 represents the total relative yields of (H_2_/D_2_)-containing complexes, which is plotted as a function of ion-trap temperature between 15 K and 200 K in Fig. 1. The formation of dihydrogen complexes is already observed at room temperature, confirming the relatively strong interaction between the Cu(i) cation and dihydrogen.^14^ Ion yields larger than ∼4% are observed for temperatures of 200 K and below. Dihydrogen adsorption increases with decreasing temperature. Moreover, at sufficiently low temperatures (<80 K) the adsorption of additional dihydrogen molecules is possible leading to the formation of *n* > 2 complexes (see Fig. S1†). The ion yields in Fig. 1 show that D_2_-containing complexes are more likely formed than the H_2_-containing complexes over the complete temperature range, revealing a substantial isotope effect in dihydrogen adsorption at Cu^+^(H_2_O). This isotopologue selectivity increases with decreasing temperature, reaching a maximum at 120 K. At lower temperatures the contribution of *n* > 2 complexes and eventually saturation of the dihydrogen binding sites needs to be considered.

### Infrared photodissociation spectra

In order to characterize the nature of H_2_/D_2_ adsorption on Cu^+^(H_2_O) in a more detailed way, we measured vibrational action spectra of the complexes using infrared photodissociation (IRPD) spectroscopy. For a comparison of the experimental IRPD spectra to computed IR spectra it proves helpful to measure the IRPD spectra in the linear absorption regime in the range of interest (2500–4500 cm^−1^), which covers the excitations of the O–H/O–D and H–H/D–D fundamental transitions. To avoid spectral overlap of the ligand-specific excitations, *e.g.*, O–H/O–D stretching transitions, we focus on the Cu^+^(D_2_O)(H_2_) and Cu^+^(H_2_O)(D_2_) complexes here.

**Fig. 2:**
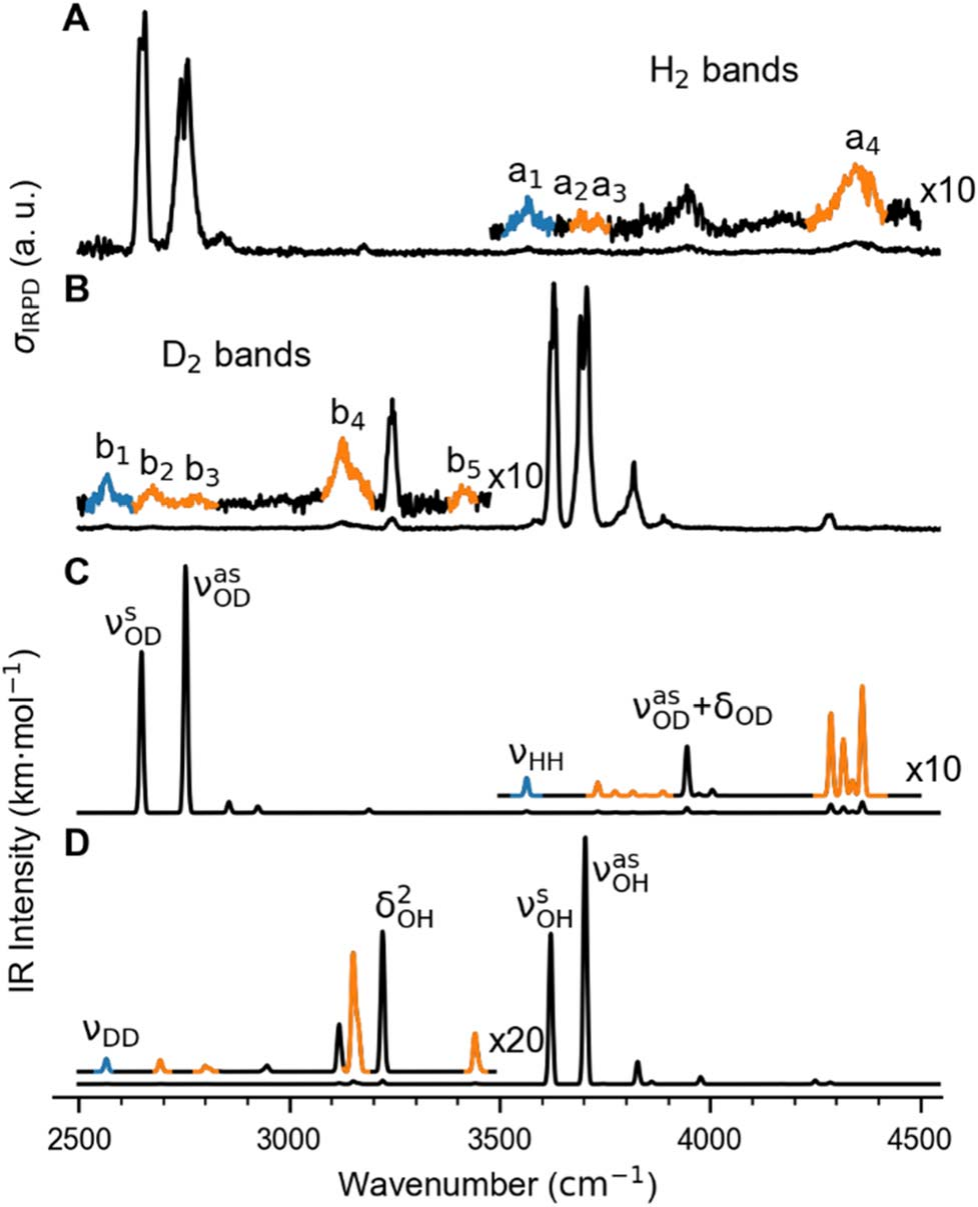
Vibrational spectra of Cu^+^(D_2_O)(H_2_)_2_ and Cu^+^(H_2_O)(D_2_)_2_. (A) and (B): Experimental IRPD spectra. (C) and (D): Predicted infrared spectra considering anharmonic contributions (VPT2/MP2/def2-TZVPP) as well as a 10 cm^−1^ wide Gaussian lineshape function. Bands related to the excitation of the dihydrogen stretching fundamentals *ν*_HH_ (a_1_) and *ν*_DD_ (b_1_), are color-coded in blue and the corresponding combination bands in orange (see Table 1 for the band assignments).

The BDE for Cu^+^(D_2_O)(H_2_) → Cu^+^(D_2_O) + H_2_ is too high to allow for efficient single-photon dissociation. Therefore the IRPD spectra of the *n* = 2 complexes, Cu^+^(D_2_O)(H_2_)_2_ and Cu^+^(H_2_O)(D_2_)_2_, were measured and these are compared to the corresponding calculated IR spectra in Fig. 2 (see Fig. S2† for the IPRD spectra of Cu^+^(H_2_O)(H_2_)_2_ and Cu^+^(D_2_O)(D_2_)_2_). The most prominent IRPD bands (see Table 1 for band positions) are assigned to the partly-resolved rovibrational transitions of the symmetric (*ν*^s^_OH/D_) and the antisymmetric O–H/O–D stretching fundamentals (*ν*^as^_OH/D_). The corresponding bands in the IRPD spectrum of Cu^+^(H_2_O)(D_2_)_2_ are observed at 3628 cm^−1^ (*ν*^s^_OH_) and 3700 cm^−1^ (*ν*^as^_OH_), which is 4 cm^−1^ higher than the previously reported values for Cu^+^(H_2_O)Ar_2_.^18^ Likewise, *ν*^s^_OD_ and *ν*^s^_OD_ for Cu^+^(D_2_O)(H_2_)_2_ are at 2651 cm^−1^ and 2751 cm^−1^, only 2 cm^−1^ different from the bands for Cu^+^(D_2_O)Ar_2_. This minor effect of dihydrogen adsorption on the O–H/O–D stretching frequencies in Cu^+^(H_2_O/D_2_O) is in line with H_2_/D_2_ binding directly to the Cu^+^ cation.

**Table 1:** Band positions (in cm^−1^) observed in the IRPD spectra of Cu^+^(D_2_O)(H_2_)_2_ and Cu^+^(H_2_O)(D_2_)_2_ shown in Fig. 1, VPT2/MP2/def2-TZVPP vibrational frequencies and intensities (in km mol^−1^ in parenthesis) and band assignments

Label	IRPD	Calculated frequency (intensity)	Assignment^a^
**Cu** ^**+**^ **(D**_**2**_ **O)(H**_**2**_ **)**_**2**_			
	2651^b^	2650 (112)	*ν* ^s^_OD_
	2751^b^	2754 (165)	*ν* ^a^_OD_
	2840	2857 (7)	*ν* ^as^_OD_ + *δ*^OOP^_OD_
	3177	3190 (2)	*ν* ^as^_OD_+ *δ*^IP^_OD_
a_1_	3568	3564 (0.7), 3563 (0.2)	*ν*_HH_
a_2_	3693	3698 (0.1)	*ν*_HH_+ *τ*^OOP^_OD_
a_3_	3733	3733 (17), 3777 (18)	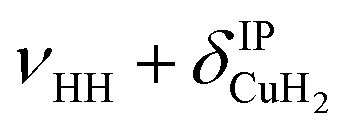
	3944	3945 (2), 4005 (0.3)	*ν* ^as^_OD_ + *δ*_DOD_
a_4_	4350	4287 (4), 4362 (5), 4316 (2), 4338 (0.7)	*ν*_HH_ + *ν*_CuH_2__

**Cu** ^**+**^ **(H**_**2**_ **O)(D**_**2**_ **)**_**2**_			
b_1_	2567	2566 (0.4), 2568 (0.1)	*ν*_DD_
b_2_	2675	2692 (0.2), 2695 (0.1)	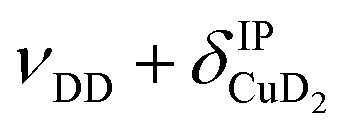
b_3_	2783	2800 (0.2), 2810 (0.1)	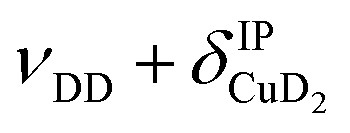 , *ν*_DD_ + *δ*_D_2_CuD_2__
b_4_	3125	3151 (0.3), 3151 (2), 3158 (1), 3165 (1)	*ν*_DD_+ *ν*_CuD_2__
	3244	3222 (3)	2 × *δ*_DOD_
b_5_	3414	3441 (0.5), 3442 (0.3), 3450 (0.1)	*ν*_DD_ + *ν*_DCuD_
	3628^b^	3622 (198)	*ν* ^s^_OH_
	3700^b^	3703 (120)	*ν* ^a^_OH_
	3819	3826 (18)	*ν* ^a^_OH_ + *τ*^OOP^_OH_
	3889, 3900	3860 (4)	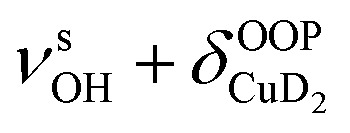
	4207	4170 (0.1)	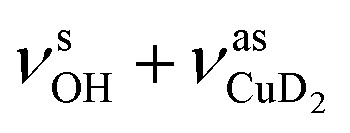
	4283	4250 (3), 4286 (1)	

a Labeling of vibrational modes: *ν* (stretching), *ν*^s^ (symmetric stretching), *ν*^as^ (antisymmetric stretching), *δ* (bending), *δ*^IP^ (in-plane bending), *δ*^OOP^ (out-of-plane bending), *τ*^OOP^ (out-of-plane hindered rotation, or libration). Only modes with IR intensities larger than 0.1 km mol^−1^ are listed.

b Values are obtained from the fitting of the rovibrational profiles (see Fig. S2).

The bands related to the excitation of H–H stretching mode (*ν*_HH_) are best observed in the IRPD spectrum of Cu^+^(D_2_O)(H_2_)_2_, shown in Fig. 2A, and labelled a_1–4_. The *ν*_HH_ fundamental (a_1_) appears at 3568 cm^−1^, substantially lower than the *ν*_HH_ fundamentals at 3729 cm^−1^ in the previously reported IRPD spectrum of Cu^+^(H_2_)_4_, indicating a stronger Cu^+^–H_2_ interaction in Cu^+^(D_2_O)(H_2_)_2_ than in Cu^+^(H_2_)_4_.^13^ Features with excitation energies above a_1_ are observed at 3693 cm^−1^ (a_2_), 3733 cm^−1^ (a_3_) and 4350 cm^−1^ (a_4_). These are tentatively assigned to combination bands (orange-coloured bands in Fig. 2B). Corresponding features are also observed in the IRPD spectrum of Cu^+^(H_2_O)(D_2_)_2_ (Fig. 2B). They are assigned to the *ν*_DD_ fundamental (b_1_, 2567 cm^−1^) as well as the combination bands at 2675 cm^−1^ (b_2_), 2783 cm^−1^ (b_3_), 3125 cm^−1^ (b_4_) and 3414 cm^−1^ (b_5_).

### Computational results

#### Structure and energetics

**Fig. 3:**
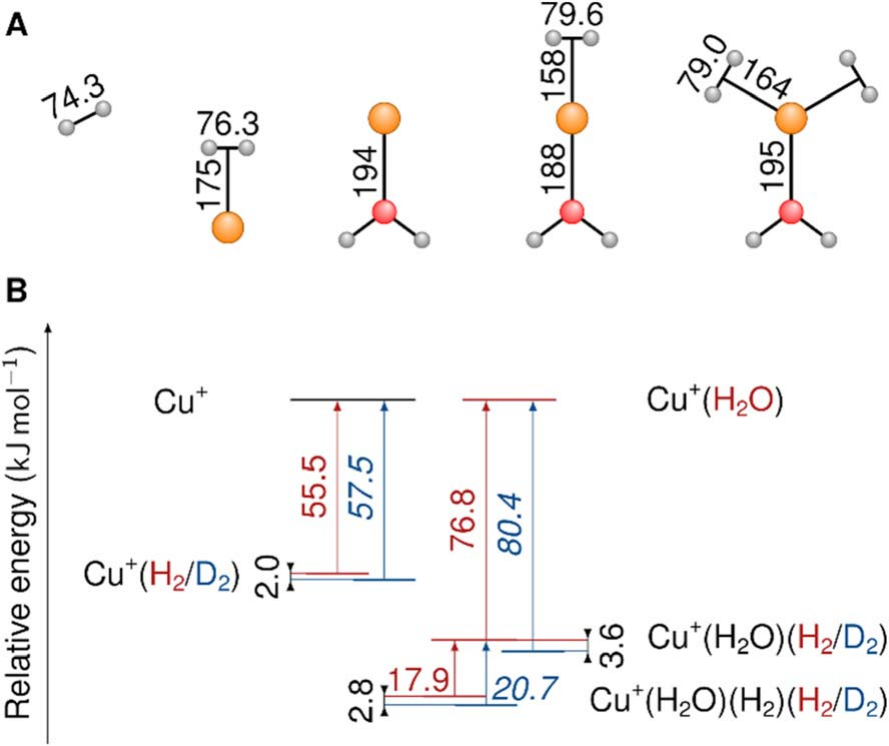
(A) MP2/def2-TZVPP bond lengths (pm) in Cu^+^(H_2_), Cu^+^(H_2_O) and Cu^+^(H_2_O)(H_2_)_1,2_ (colours: Cu orange, O red, H grey). (B) MP2/def2-TZVPP dissociation energies (kJ mol^−1^) with anharmonic (VPT2) ZPE correction. The red values (blue values in italic font) represent the dissociation energies of H_2_ (D_2_). Dissociation ZPE differences are highlighted.

MP2/def2-TZVPP minimum-energy structures and BDEs of Cu^+^(H_2_O)_0,1_(H_2_/D_2_)_0–2_ are shown in Fig. 3. The global minimum-energy structures of Cu^+^(H_2_O)(H_2_)_1,2_ are of C_2v_ symmetry. Both hydrogen molecules bind directly to the Cu^+^ ion. A single H_2_ ligand binds to Cu^+^(H_2_O) remarkably strongly with a BDE (D_0_) of 76.8 kJ mol^−1^, which is considerably higher than the 55.5 kJ mol^−1^ found for one H_2_ bound to the bare Cu^+^ ion. This underscores the role of the *trans*-positioned water molecule in increasing the bond strength between Cu^+^ and H_2_. However, only 17.9 kJ mol^−1^ is gained when binding a second H_2_, which is in line with a higher Cu^+^–H_2_ distance of 1.64 Å for the two H_2_ molecules in Cu^+^(H_2_O)(H_2_)_2_ (compared to 1.58 Å in the complex with one H_2_). We will revisit the effect of H_2_ adsorption on the ion-ligand bond lengths in the bonding analysis section. The addition of a third H_2_ molecule yields only 2.37 kJ mol^−1^.

When compared to the bond length in the free hydrogen molecule, the H–H distance in the Cu^+^(H_2_O)(H_2_) and Cu^+^(H_2_O)(H_2_)_2_ complexes is elongated by 5.3 pm and 4.7 pm, respectively (see Table S6†). This activation of the H–H (D–D) bond is substantially larger than the 2.3–3.1 pm reported for Cu(H_2_)_4_^+^ and is in line with the much stronger redshift of the H–H (D–D) stretching fundamental observed for the present system.

#### Vibrational spectra and band assignments

In order to assign the bands observed in the IRPD spectra, we calculated IR spectra including anharmonic contributions using vibrational perturbation theory (see computational section for details of the VPT2/MP2/def2-TZVPP calculations). The IR spectra for Cu^+^(D_2_O)(H_2_)_2_ and Cu^+^(H_2_O)(D_2_)_2_ (shown in Fig. 2C and D) are determined from VPT2 frequencies and intensities (listed in Table 1) using a 10 cm^−1^ wide Gaussian lineshape function.

Only the VPT2 IR bands involving excitation of H_2_ or D_2_ stretching modes, either as a fundamental (blue bands) or as part of a combination transition (orange bands), are color-coded. Based on the agreement between the experimental and computed vibrational spectra shown in Fig. 2, we can unambiguously assign all the observed bands (see Table 2). The excitation of the H–H (D–D) stretching fundamental a_1_ (b_1_) is observed at *ν*_HH_ = 3568 cm^−1^ (*ν*_DD_ = 2567 cm^−1^), resulting in a frequency ratio *ν*_HH_/*ν*_DD_ of 1.390. This ratio is close to the value of 1.388 obtained from the VPT2 IR spectra, confirming the high quality of the MP2/def2-TZVPP potential along the vibrational mode. The combination bands a_2_ to a_4_, observed at 3693 cm^−1^, 3733 cm^−1^, and 4350 cm^−1^, respectively, are assigned to combined excitations of the H–H stretching modes (*ν*_HH_) with either of the following low-frequency vibrational modes: out-of-plane OD hindered rotation (*τ*^OOP^_OD_), in-plane Cu^+^–H_2_ bending (
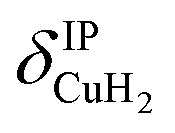
), and Cu^+^–H_2_ stretching modes (*ν*_CuH2_). For the latter two modes, the present assignment is similar to the one made previously for the IRPD spectrum of Cu^+^(H_2_)_4_.^13^ Analogously, the combination bands b_2_ to b_5_, observed at 2675 cm^−1^, 2783 cm^−1^, 3125 cm^−1^ and 3414 cm^−1^, respectively, are assigned to combination excitations of one of the two D–D stretching modes (*ν*_DD_) with Cu^+^–D_2_ bending (
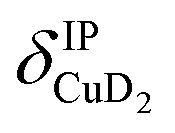
 and 
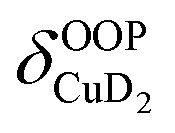
) or the Cu^+^–D_2_ stretching excitations (
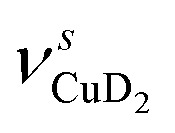
 and *ν*_DCuD_).

**Table 2:** EDA-NOCV result for Cu–H_2_ bond of Cu^+^(H_2_)_2_(H_2_O), Cu^+^(H_2_)(H_2_O) and Cu^+^(H_2_)^a^

	Cu^+^(H_2_O)(H_2_)-(H_2_)	Cu^+^(H_2_O)-(H_2_)	Cu^+^-(H_2_)
Δ*E*_int_	−71	−112	−95
Δ*E*_int_(disp)^b^	−3(4%)	−2(2%)	−2(2%)
Δ*E*_int_(elec)^b^	−68(96%)	−110(98%)	−93(98%)
Δ*E*_Pauli_	193	188	189
Δ*E*_elstat_^c^	−151(58%)	−172(58%)	−155(55%)
Δ*E*_orb_^c^	−110(42%)	−126(42%)	−127(45%)
Δ*E*_1_(σ(H_2_)→ Cu(d))^d^	−58(53%)	−79(63%)	−69(54%)
Δ*E*_2_(Cu(d)→ σ*(H_2_)) d	−24(22%)	−19(15%)	−38(30%)
Δ*E*_3_(Cu(d)→ σ*(H_2_)) d	−26(24%)	−25(20%)	−18(14%)
Δ*E*_prep_	44	5	4
Δ*E*_prep_(H_2_)	4	5	4
Δ*E*_prep_(Cu^+^L_0–3_)	40	0	0
Δ*E*_bond_	−27	−107	−91
*r*(Cu–H_2_)^e^	1.72	1.64	1.70

a All energies in kJ mol^−1^, bond distances in Å computed with B3LYP-D4/TZ2P. Fragments are closed-shell species Cu^+^L_0–3_ and H_2_ (L = H_2_O, H_2_).

b Percentage values give the relative contributions of dispersion and electronic effects to Δ*E*_int_.

c Percentage values give the relative contributions to the attractive pEDA terms Δ*E*_elstat_ and Δ*E*_orb_.

d Percentage values give the relative contributions of the NOCV to Δ*E*_orb_.

e Distance between Cu and the centre of the H_2_ fragment.

The remarkably good agreement between the IRPD and VPT2 spectra in Fig. 2, which is discussed in detail in the ESI,† confirms that the chosen computational method provides an adequate description of also the low-frequency M^+^–H_2_/D_2_ vibrational modes, which play an important role in modelling the isotopologue selectivity. However, this appears to be in part due to a favourable error compensation between, *i.e.*, the neglect of substantial relativistic effects, basis set insufficiencies, and the overestimation of London dispersion interaction inherent in the MP2 method (see ESI†).

#### Bonding analysis of Cu^+^–H_2_ and Cu^+^–H_2_O bonds

The nature of the Cu^+^–H_2_ and Cu^+^–H_2_O interactions in the complexes investigated was analysed with an Energy Decomposition Analysis^19–23^ together with the Natural Orbital for Chemical Valence approach (EDA-NOCV)^20–24^ method. EDA allows the decomposition of the bonding energy Δ*E*_bond_ between fragments, which correspond to negative dissociation energy, into chemically meaningful contributions. The bonding energy Δ*E*_bond_ is represented as a sum of interaction energy Δ*E*_int_ and preparation energy Δ*E*_prep_ where the latter describes the deformation of the fragments during the bond formation. The interaction energy Δ*E*_int_ can be further split into dispersion Δ*E*_int_(disp) and electronic effects Δ*E*_int_(elec). The electronic effects can then be divided into quasi-electrostatic contribution Δ*E*_elec_, Pauli repulsion Δ*E*_Pauli_ and orbital interaction Δ*E*_orb_. The NOCV extension helps to find the most important orbital interactions to the bond as further decomposition of Δ*E*_orb_.

**Fig. 4:**
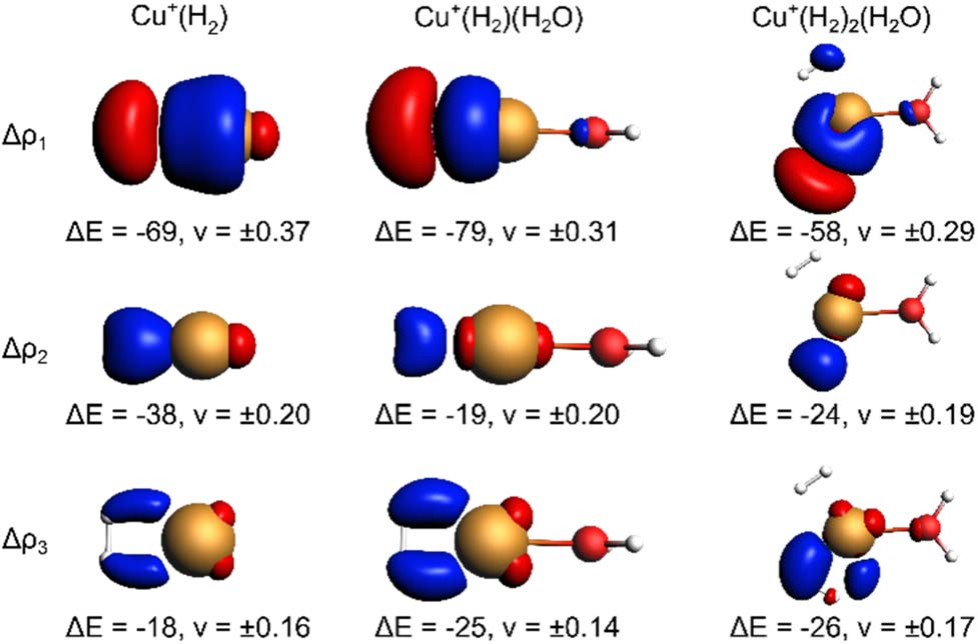
Selected deformation densities (Δ*ρ*_i_) from EDA-NOCVs for Cu–H_2_ bond with energy contribution (Δ*E*_i_) to Δ*E*_orb_ in kJ mol^−1^ and eigenvalues (*ν*_i_) Charge depletion (red) and charge accumulation (blue) with isosurface value 0.0025 for Cu(H_2_)^+^, 0.0020 for Cu(H_2_)(H_2_O)^+^ and 0.0012 for Cu(H_2_)_2_(H_2_O)^+^.

The EDA-NOCV results for the complexes investigated in this work are shown in Table 2. They confirm a donor–acceptor-type interaction in the Cu^+^–H_2_ bond in all investigated complexes (Cu^+^(H_2_O)(H_2_)_2_, Cu^+^(H_2_O)(H_2_), and Cu^+^(H_2_)). At first glance, the stronger bonding in Cu^+^(H_2_O)(H_2_) compared to Cu^+^(H_2_) is due to an increase in the electrostatic attraction (difference ΔΔ*E*_elstat_ = −17 kJ mol^−1^) while the orbital interaction (Δ*E*_orb_) and Pauli repulsion (Δ*E*_Pauli_) do not change significantly. However, a closer look reveals a more intriguing trend. The bond in Cu^+^(H_2_) is significantly longer compared to Cu^+^(H_2_O)(H_2_). If we analyse the main deformation densities (Fig. 4), we find that upon coordination of H_2_O, the σ-donation from σ(H_2_) into the d-orbitals at Cu (Δ*E*_1_) increases by 10 kJ mol^−1^. But the in-plane back-bonding (Δ*E*_2_) decreases by a larger amount (−19 kJ mol^−1^), while the out-of-plane back-bonding (Δ*E*_3_) is slightly increased (+7 kJ mol^−1^). This reduced in-plane back-bonding is due to the involvement of the Cu(d)-orbitals in the bonding to the H_2_O ligand, which can be seen in the deformation densities Δ*ρ*_1_ and Δ*ρ*_2_ in Fig. 4. This leads to a lower Pauli repulsion and allows the H_2_ molecule to bind closer to the Cu centre. While orbital attraction and Pauli repulsion thus balance at the same values compared to Cu^+^(H_2_), the electrostatic attraction is increased. The main reason is thus lowering of Pauli repulsion leading to a shorter Cu–H_2_ distance in Cu^+^(H_2_O)(H_2_).

The weakening of the Cu^+^–H_2_ bond in Cu^+^(H_2_O)(H_2_)_2_ compared to Cu^+^(H_2_O)(H_2_) is attributed mainly to the large preparation energy resulting from the transition from a linear to a trigonal structure. Additionally, the larger bond lengths are due to the stronger Pauli repulsion between the higher number of ligands and contribute to this phenomenon because they weaken the attractive interactions compared to Cu^+^(H_2_O)(H_2_).

Note that the Cu^+^–H_2_O bond is stronger in Cu^+^(H_2_O)(H_2_) compared to Cu^+^(H_2_O), which is mainly due to reduced Pauli repulsion as an effect of the back-bonding contributions from Cu^+^ to H_2_ (see NOCV in Fig. S9 in ESI†). Adding another H_2_ molecule, *i.e.* the formation of Cu^+^(H_2_O)(H_2_)_2_, leads to a weaker Cu^+^–H_2_O bond due to competition of the three ligands for the electrons at the central atom.

### Anharmonic contribution to the BDEs for different isotopologues

As shown in Table 3, going from Cu^+^(H_2_O)(H_2_)_2_ to Cu^+^(H_2_O)(T_2_)_2_, the BDE of the H_2_ isotopologue increases due to the increasing mass of the isotopologues and the consequently lower ZPE. The difference in the adsorption ZPE between the D_2_ and the H_2_ complexes is 2.8 kJ mol^−1^ (1.0 kJ mol^−1^ between T_2_ and D_2_), which is slightly larger than the one observed in Cu(i)-MFU-4l (2.5 kJ mol^−1^) by I. Weinrauch *et al.*^4^ and about one-third greater than the ZPE change for the exchange reaction Cu^+^(D_2_)_3_(H_2_) + D_2_ → Cu^+^(D_2_)_4_ + H_2_ (2.1 kJ mol^−1^) observed in the previous study conducted by some of us.^13^

**Table 3:** Calculated CCSD(T)/aug-cc-pVTZ(-PP) BDEs (*D*_0_) including the desorption ZPE (*Δ*_des_ZPE) calculated using VPT2 on the MP2/def2-TZVPP potential energy surface; anharmonic contribution (VPT2 level) for the dissociation of H_2_ isotopologues, *i.e.* Cu^+^(H_2_O)(H_2_)_2_ → Cu^+^(H_2_O)(H_2_) + H_2_. All values reported in kJ mol^−1^

Species	*D*_0_	*Δ*_des_ZPE	Anharmonic contribution
Cu^+^(H_2_O)(H_2_)_2_	16.9	−10.8	0.8
Cu^+^(H_2_O)(D_2_)_2_	19.7	−8.0	0.4
Cu^+^(H_2_O)(T_2_)_2_	21.0	−6.7	0.3
Cu^+^(H_2_O)(HD)_2_	18.4	−9.3	0.6
Cu^+^(H_2_O)(HT)_2_	19.2	−8.5	0.5
Cu^+^(H_2_O)(DT)_2_	20.4	−7.3	0.3

Our calculations also show that the anharmonic contribution to the H_2_ desorption ZPE for the Cu^+^(H_2_O)(H_2_)_2_ → Cu^+^(H_2_O)(H_2_) + H_2_ reaction is more than three times as big as the value obtained for free H_2_ (0.2 kJ mol^−1^). Similar factors are observed for D_2_ (3.1) and T_2_ (3.1). This demonstrates the substantial influence of anharmonic contributions on the thermodynamic equilibrium of the isotopologue exchange reaction, reducing, *e.g.*, the energy difference between H_2_ and D_2_ adsorption by 12%. As expected, the anharmonic contributions to the vibrational frequencies and ZPEs are inversely proportional to the reduced mass of the dihydrogen isotopologue.

### H_2_ isotopologue selectivity

**Fig. 5:**
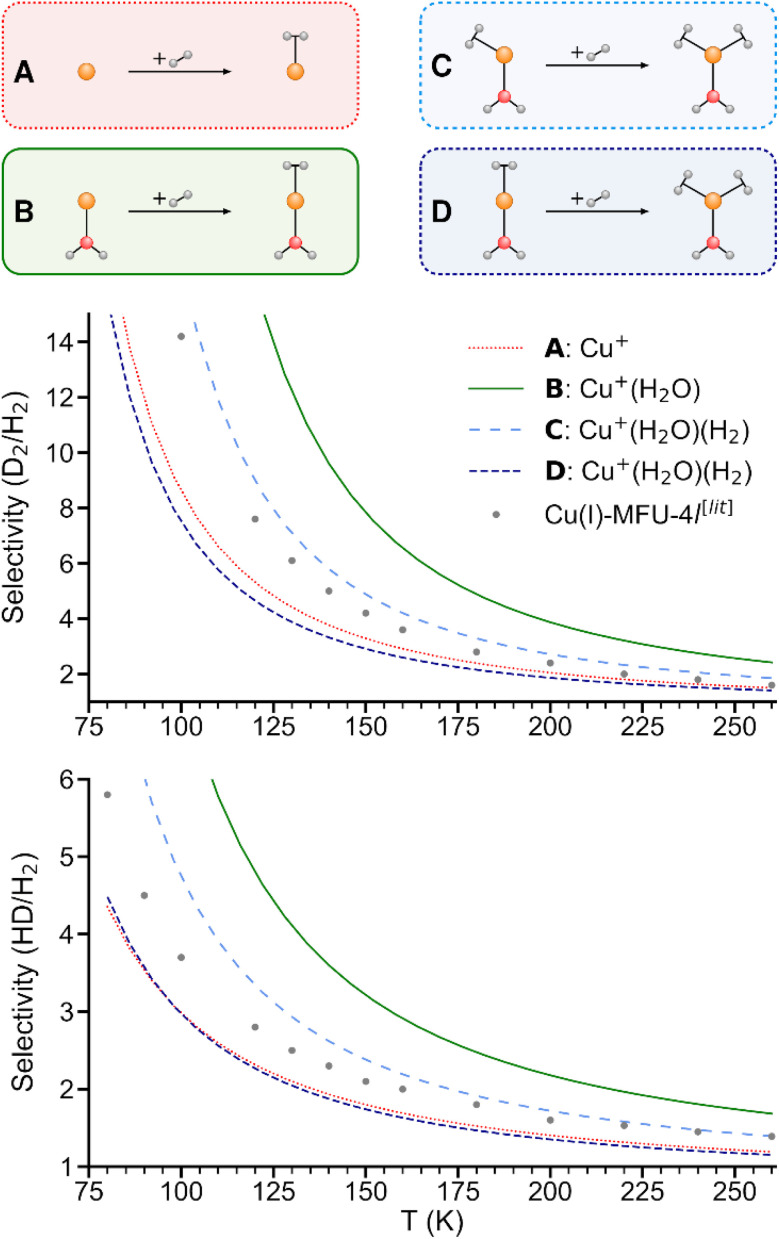
Predicted selectivities for dihydrogen isotopologue adsorption at Cu^+^, Cu^+^(H_2_O), and Cu^+^(H_2_O)(H_2_) as a function of temperature. For the latter, two models are compared (see text). Literature data of Cu(i)-MFU-4l are shown for comparison.^4^

As shown in Fig. 5, the predicted isotopologue selectivity of H_2_ adsorption is much higher at Cu^+^(H_2_O) (B) than at Cu^+^ (A) and decreases again for a second H_2_ adsorbing at Cu^+^(H_2_O)(H_2_). We study two scenarios for the resulting Cu^+^(H_2_O)(H_2_)_2_ complex: in the first one, we assume that the second H_2_ molecule adsorbs at the free space of a hypothetical Cu^+^(H_2_O)(H_2_) complex where H_2_O and the first H_2_ are already positioned as in Cu^+^(H_2_O)(H_2_)_2_, thus mimicking a scenario as it could occur on an undercoordinated adsorption site in a porous solid (C). Here, only the six degrees of freedom of the considered H_2_ molecule are taken into account to calculate the isotopologue selectivity of dihydrogen adsorption, an approach that has been used in previous studies of MOFs.^4^

In the second one (D), all complexes are fully optimized and the degrees of freedom of all involved H_2_ moieties are considered, which can be compared directly with the IRPD experiments. As a consequence of the weaker binding energy per H_2_ molecule, the ZPE per H_2_ and finally the overall selectivity is reduced significantly.

We conclude that a model with a rigid ligand structure is insufficient when dealing with structurally flexible adsorption sites such as undercoordinated metal ions, as it will lead to spurious results due to ligand reorientation. Conversely, as evidenced by prior studies,^4,8^ rigid adsorption sites with structurally constraining ligands like in the hypothetical starting structure in Fig. 5C may be essential for achieving a high adsorption energy and isotopologue selectivity.

## Summary and conclusions

We elucidated the strong hydrogen isotopologue selectivity of undercoordinated Cu(i) sites by examining the model complexes Cu^+^(H_2_O)(H_2_)_*n*_ (with *n* = 0, 1, 2) using experiment and theory. Both Cu^+^(H_2_O) and Cu^+^(H_2_O)(H_2_/D_2_) show an obvious isotope effect in the adsorption of H_2_ and D_2._ The observed vibrational spectra of Cu^+^(H_2_O)(H_2_)_2_ and its isotopologues in the range from 2500–4500 cm^−1^ match the calculated frequencies of the fundamentals and related combination bands of H_2_/D_2_ when taking anharmonicity into account. This suggests that these systems are ideal model complexes for gas-phase studies of the chemistry at individual active sites as they occur in framework materials. The stronger H_2_ affinity of Cu(i) coordinated by a single oxygen-donor ligand, in comparison to the bare cation, is explained by EDA and traced back to lowering of Pauli repulsion which allows the H_2_ molecule to bind closer to the Cu centre. Finally, we used the computational data to predict the dihydrogen isotopologue selectivity of the formation of Cu^+^(H_2_O)(H_2_)_*n*_ complexes, observing a significant selectivity decrease for *n* = 2 *vs. n* = 1. The present study underlines that the structural environment of undercoordinated metal centres in framework materials play a crucial role in the local chemistry and demonstrate particular promise for rigid frameworks with highly exposed metal sites.

## Methods

### Experimental methods

The experiments were performed on the Leipzig 5 K ring-electrode ion-trap triple mass spectrometer described previously.^16^ Cu^+^(H_2_O) cations are transferred to the gas phase from a 5 mmol CuSO_4_ (Sigma Aldrich: CuSO_4_·5H_2_O 99% in CH_3_OH 99.9% and distilled water) solution using a nanospray ion source under open atmospheric conditions, while the Cu^+^(D_2_O) cations are produced *via* H/D exchange in moist air conditions saturated with heavy water vapour (99.9% D_2_O). The beam of cations is skimmed, thermalized to room temperature in a Helium-filled radio-frequency ion guide, and then mass-selected in a quadrupole mass filter. Mass-selected Cu^+^(H_2_O/D_2_O) ions are continuously trapped in a radio-frequency ring-electrode ion trap, held at a temperature in-between 15 K and 295 K using a closed-cycle helium cryostat and filled with ≈ 1 mbar H_2_(D_2_). Cu^+^(H_2_O/D_2_O)(H_2_/D_2_)_*n*_ adducts are formed by three-body collisions and thermalized to the ambient temperature by many collisions with other gas molecules.

All cations are extracted from the ion trap every 100 ms and weakly focused both temporally and spatially into the centre of the extraction region of an orthogonally mounted double-focusing reflectron time-of-flight (TOF) mass spectrometer. For the determination of the ion yields, the ions are extracted and accelerated without using the reflectron. Furthermore, TOF mass spectra are recorded for TOF extraction delay times ranging from 100 μs to 135 μs and summed to account for incomplete temporal focusing of the extracted ions. The absolute ion yield *I*_j_ of the j'th ion is obtained by integration of the corresponding TOF peak, which is assumed proportional to the number of detected cations of a particular mass-to-charge ratio. Multiple TOF mass spectra are measured and averaged to determine the relative ion yield 
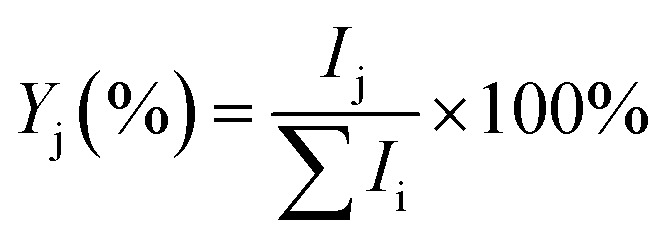
.

IRPD spectra are measured using the IR^1^MS^2^ scheme.^25^ To this end, the cation packet extracted from the ion trap is accelerated into the reflectron stage and refocused in the initial extraction region. Prior to reacceleration towards the MCP detector, ions with a particular mass-to-charge ratio are selectively irradiated with a properly timed, widely wavelength tunable (750–7000 cm^−1^) IR laser pulse (bandwidth: ∼3.5 cm^−1^), supplied by an optical parametric oscillator/amplifier (LaserVision) laser system.^26^ IRPD spectra are recorded by continuously scanning the laser wavelength that is monitored online using a HighFinesse WS6-600 wavelength meter. The scan speed is set to obtain an averaged TOF mass spectrum over 40–80 laser shots every 2 cm^−1^. Typically, four scans are measured and averaged to obtain each IRPD spectrum. The photodissociation cross section *σ*_IRPD_ determined as described previously.^16,27^

### Computational methods

Structure optimizations and single point energy calculations were performed using coupled cluster theory with single, double, and perturbative triple excitations, *i.e.* CCSD(T), utilizing CFOUR 2.1.^28^ The heavily augmented correlation-consistent aug-cc-pVTZ basis set was used for all atoms except for copper, where the version with an effective core potential (ECP), *i.e.* aug-cc-pVTZ-PP, was used to take into consideration relativistic effects. The combination will be referred to as “aug-cc-pVTZ(-PP)” henceforth. Very tight SCF convergence criteria for the Hartree–Fock equations and the coupled cluster amplitudes equations were used (10^−10^ hartree, *i.e.* SCF_CONV = 10 and CC_CONV = 10). The geometry optimizations were performed using analytically evaluated gradients with good starting geometry in Z-matrix files and tighter thresholds. During the geometry optimizations, the RMS gradients were converged to 10^−9^ hartree.

Vibrational analysis (calculation of vibrational frequencies and intensities as well as ZPEs) was performed using standard second-order vibrational perturbation theory (VPT2) without any resonance treatment on a potential energy surface obtained with second-order Møller–Plesset perturbation theory (MP2) in conjunction with the def2-TZVPP basis set using the Gaussian^29^ program package. For the preceding geometry optimization, internal coordinates and very tight geometry convergence criteria were used. The frozen-core approximation was applied throughout by specifying five orbitals (DROPMO = 1 > 5), *i.e.* freezing the 1s, 2s, and the three 2p orbitals of the Cu atom. The full details of the computed structures for Cu^+^(H_2_O)(H_2_)_0,1,2_ complexes are given in the ESI.†

For calculation of dihydrogen isotopologue selectivities, harmonic vibrational frequencies obtained with the Gaussian program package were used and two approaches are contrasted. One, which was used for all systems considered, follows a previous methodology,^4,7^ which relies on the calculation of the partition functions for the individual contributions of each of the six normal modes of H_2_ in the field of the frozen adsorption site structure. Here, we used the fragment structure of the fully optimized complex. The harmonic approximation was applied to analyse five of the six modes (*ν*_HH_, *ν*_CuH_2__, 
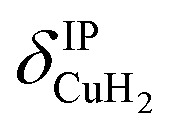
, 
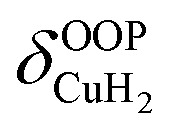
, 
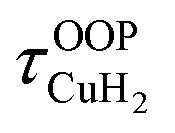
) of the adsorbed hydrogen isotopologues, which are visualized in Fig. S6† (see ESI†). The sixth mode, *i.e.* the quasi-free rotation of H_2_ about the Cu–H_2_ axis, was treated using the model of the rigid rotor rotating in the plane. For Cu^+^(H_2_O)(H_2_)_2_, this was contrasted with a second approach, which uses fully relaxed structures and considers the change of the vibrational properties of both H_2_ upon binding of the second, *i.e.* considering twelve vibrational modes in Cu^+^(H_2_O)(H_2_)_2_, six in Cu^+^(H_2_O)(H_2_) and the six in free H_2_.

The bonding analysis with the EDA-NOCV analysis, was performed using the Amsterdam Modeling Suite (AMS, version 2021.105),^30^ where complexes and their fragments are optimized beforehand. All DFT calculations, were carried out using the B3LYP functional^31^ with D4 dispersion correction of Grimme^32^ and TZ2P basis set.^33^ The selection of the xc-functional is based on the benchmark study presented in ESI.† Relativistic effects were considered using the zeroth-order regular approximation (ZORA).^34^ Symmetry was not considered. The SCF convergence criterion was set to 10^−6^ E_h_, indicated by the keyword ‘Very Good’ as a numerical quality indicator. Geometry optimization accounted for the energy criterion at 3 × 10^−3^ E_h_ and the gradient criterion at 10^−3^ E_h_ Å^−1^.

## Data availability

The data that support the findings of this study are openly available in zenodo at https://doi.org/10.5281/zenodo.12554684.

## Author contributions

EGD and SH contributed equally. SH and JJ performed the experiments. EGD performed the CCSD(T) and MP2 calculations, vibrational analysis and the selectivity calculations. FK carried out the bonding analysis. TH, KA and RTZ conceived the original idea of the project. All authors provided feedback and contributed to the final version of the manuscript.

## Conflicts of interest

There are no conflicts to declare.

## Supplementary Material

SC-015-D4SC04582C-s001
