# Supplementary material for: Direct evidence for ligand-enhanced activity of Cu(i) sites
Source: Chem Sci. 2024 Aug 16;15(36):14635–43. doi: 10.1039/d4sc04582c (PMC11460435; doi:10.1039/d4sc04582c)
Supplement: SC-015-D4SC04582C-s001 [file SC-015-D4SC04582C-s001.pdf]

Supplementary Information (SI) for Chemical Science.
This journal is © The Royal Society of Chemistry 2024

Supplementary Information

Direct evidence for ligand-enhanced activity of Cu(I) sites

Elvira Gouatieu Dongmo[+,1,2] Shabnam Haque[+,1] Florian Kreuter,[1] Toshiki Wulf,[1,3] Jiaye Jin,[1] Ralf Tonner-Zech,*[1] Thomas Heine,[*,2,3,4] and Knut R. Asmis*[1]*

1) Wilhelm-Ostwald-Institut für Physikalische und Theoretische Chemie, Universität Leipzig, Linnéstr. 2, 04103 Leipzig, Germany

2) Institute of Resource Ecology, Research Site Leipzig, Helmholtz-Zentrum Dresden-Rossendorf, Permoserstr. 15, 04318 Leipzig, Germany

3) Faculty of Chemistry and Food Chemistry, School of Science, TU Dresden, 01062 Dresden, Germany

4) Department of Chemistry and ibs for nanomedicine, Yonsei University, Seodaemun-gu, Seoul 120-749, Republic of Korea

Electronic mail: jiaye.jin@uni-leipzig.de, ralf.tonner@uni-leipzig.de, thomas.heine@tu-dresden.de, knut.asmis@uni-leipzig.de

Table of contents

TOF mass spectra 2

IRPD spectra 3

Supplementary Note 1: Validation 4

Supplementary Note 2: Influence of relativistic effects 5

Energy decomposition analysis (EDA) of the Cu^+-H_2 bond 13

TOF mass spectra

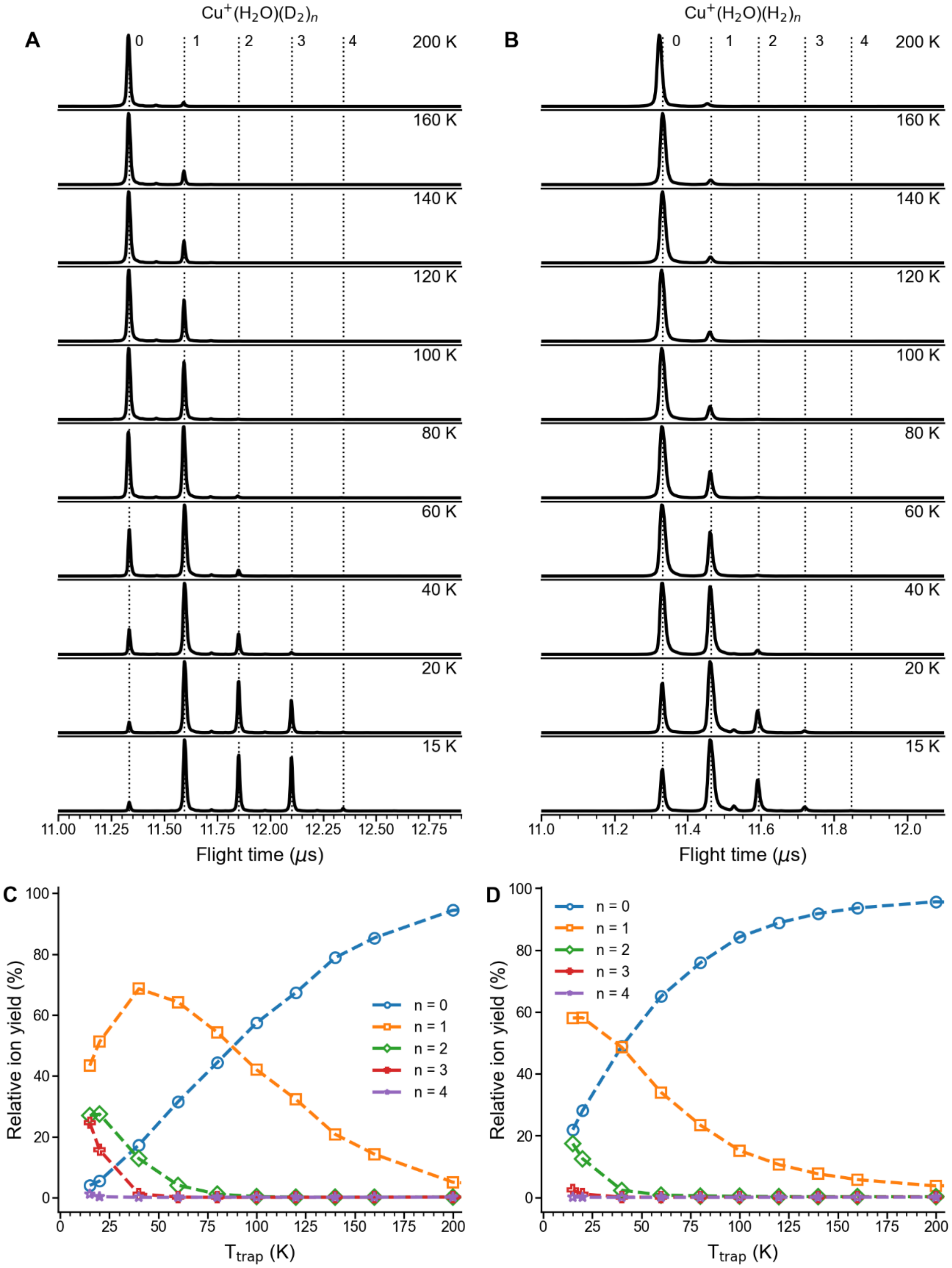

Figure S1: TOF mass spectra of (A) $Cu^+(H_2O)(D_2)_n$ and (B) $Cu^+(H_2O)(H_2)_n$ obtained after storing $Cu^+(H_2O)$ on average 50 ms in the ion trap (filled with around 1 mbar D_2 or H_2) for different ion-trap temperatures. Relative ion yield of (C) $Cu^+(H_2O)(D_2)_n$ and (D) $Cu^+(H_2O)(H_2)_n$.

IRPD spectra

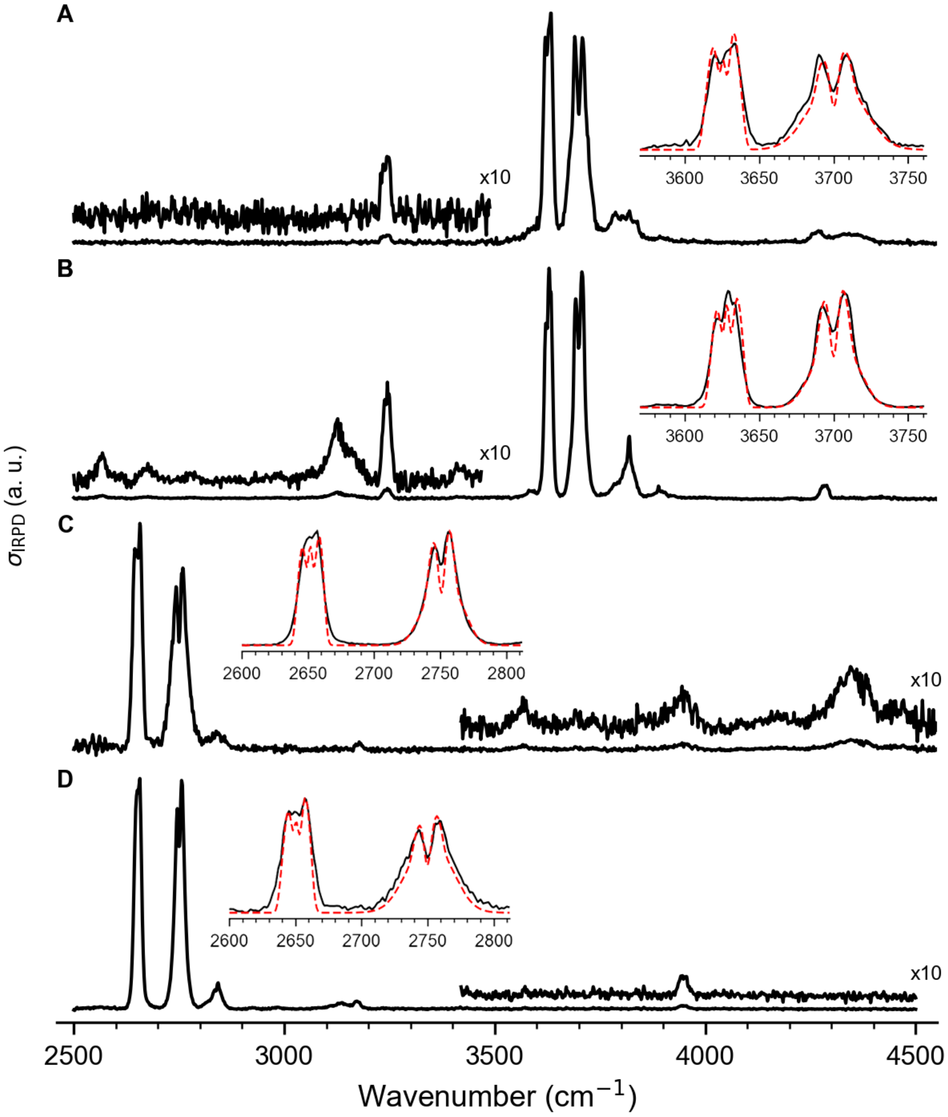

Figure S2: IRPD spectra of $Cu^{+}(H_2O)(H_2)_2$ and its isotopologues. A) $Cu^{+}(H_2O)(H_2)_2$, B) $Cu^{+}(H_2O)(D_2)_2$, C) $Cu^{+}(D_2O)(H_2)_2$, and D) $Cu^{+}(D_2O)(D_2)_2$. The spectra are magnified for showing the rovibrational profiles of OH/OD stretching bands. The rotational constants calculated using VPT2/MP2/def2-TZVPP (see following calculation results) are applied to fit the $\nu^{s}_{OH/D}$ and $\nu^{as}_{OH/D}$ frequencies, which are simulated at 100 K temperature and with 4.5 cm^{-1} width for the Gaussian lineshape profile, using the PGOPHER program.[36] The standard deviations of the fitting frequencies are 0.3 – 0.5 cm^{-1}.

Supplementary Note 1: Validation

Our calculations show that MP2/def2-TZVPP structures and harmonic frequencies (see **Table S3, Table S4** and **Figure S4**) match the CCSD(T)/aug-cc-pVTZ(-PP) ones very closely. Hence, MP2/def2-TZVPP appears to be a sufficient level of theory for the computation of anharmonic frequencies of $Cu^+(H_2O)(H_2)_2$ and its isotopoloues. The ZPE-corrected bond dissociation energy (BDE, D_0) of $Cu^+(H_2O)$ obtained using MP2/def2-TZVPP and CCSD(T)/aug-cc-pVTZ(-PP) calculations is 157 and 161 $kJ \cdot mol^{-1}$, respectively. This is in agreement with the experimental value (157 $kJ \cdot mol^{-1}$) reported by Paul R. Kemper *et al.*[14] and by previous MP2 and CCSD(T) calculations.[15,19] The calculated vibrational frequencies of H_2O and $Cu^+(H_2O)$ isotopologues are reported in **Table S7**.

Table S3. Bond lengths and angles according to CCSD(T)/aug-cc-pVTZ(-PP) (in parentheses: MP2/def2-TZVPP) calculations. Structures are shown in **Figure 3.** All interatomic distances are given in Angstrom (Å) and angles in degrees (°).

	**Symmetry**	**r(Cu-O)**	**r(O-H)**	**r(Cu-H₂)**	**r(H-H)**	**∠(H-O-Cu)**	**∠(H-O-H)**
**H_2**	C_{2v}				0.743(0.737)		
**H_2O**	C_{2v}	/	0.962(0.958)	/	/	/	104.2 (103.8)
**$Cu^+(H_2)$**	C_{2v}	/	/	1.754(1.644)	0.763(0.780)	/	/
**$Cu^+(H_2O)$**	C_{2v}	1.935 (1.931)	0.929 (0.963)	/	/	127.61 (126.47)	104.8 (107.1)
**$Cu^+(H_2O)(H_2)$**	C_{2v}	1.881(1.875)	0.965 (0.963)	1.579 (1.549)	0.796 (0.796)	125.97 (125.92)	108.0 (107.8)
**$Cu^+(H_2O)(H_2)_2$**	C_{2v}	1.948 (1.947)	0.964 (0.963)	1.642 (1.651)	0.790 (0.789)	126.40 (126.53)	107.1 (106.9)
**$Cu^+(H_2O)(H_2)_3$**	C_{2v}	(2.069)	(0.962)	(1.720)	(0.778)	(126.92)	(106.2)

Table S4. Geometrical parameters of $Cu^+(H_2O)(H_2)_2$ at different computational levels. Distances in Angstroms (Å), angles and dihedral angles in degree (°), the numbering of the atoms is given in **Figure S4.**

Parameters	CCSD(T)/ aug-cc-pVTZ (-PP) [CCSD(T)/aug-cc-pVTZ]	MP2/ def2-TZVPP	**MP2/ aug-cc- pVTZ (-PP)** [MP2/aug-cc- pVTZ]
r(Cu-O)	1.948 [1.971]	1.947	1.914 [1.938]
r(Cu-X1/X2)	1.642 [1.695]	1.651	1.591 [1.675]
r(O-H1/H2)	0.964 [0.964]	0.963	0.965 [0.965]
r(H3-H4)	0.790 [0.782]	0.789	0.802 [0.7901]
∠(H1-O-H2)	107.15 [106.90]	106.93	107.26 [106.94]
∠(H2-O-Cu)	126.43 [126.55]	126.53	126.37 [126.53]
∠(X1/X2-Cu-O)	120.64 [121.12]	119.98	120.04 [119.05]

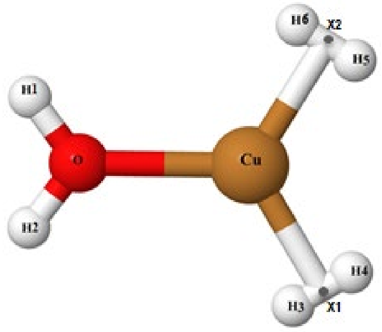

Figure S4. Optimized geometry of $Cu^+(H_2O)(H_2)_2$ at the CCSD(T)/aug-cc-pVTZ(-PP) level and definition of geometry parameters. X1 and X2 represent the centers of mass of H_2 ligands.

Supplementary Note 2: Influence of relativistic effects

The interatomic distances around the Cu^+ are substantially influenced by relativistic effects. CCSD(T) in combination with aug-cc-pVTZ(-PP), which includes relativistic effects implicitly via the effective core potentials (ECP), yields Cu–H_2 and Cu–O bonds that are respectively shorter by 3.4 pm and 2.1 pm than those calculated employing the respective all-electron basis set (see Table S4). This leads to a blue-shift of the corresponding stretching frequencies, elongation of the H–H bonds and a red-shift of the H–H stretching frequency. That this is a result of relativistic effects is corroborated by the fact that a comparison between non-relativistic DFT calculations and ones including relativistic effects employing the zeroth-order regular approximation (ZORA) yields similar results (see **Table S5**; calculations performed with AMS version 2022.104). MP2 in combination with the aug-cc-pVTZ(-PP) basis set yields even shorter bonds with commensurate effects on the respective vibrational frequencies. This is likely due to the known tendency of MP2 to overestimate London dispersion – an effect that may, to some extent, be cured by the use of a moderately sized basis set that is too small to capture the full strength of the partially spurious MP2 dispersion. It is likely for this reason that (as shown in **Table S6**) MP2 in combination with the moderately sized def2-TZVPP basis set gives bonds lengths and harmonic frequencies very similar to the CCSD(T)/aug-cc-pVTZ(-PP) benchmark. The remarkably good agreement between the experimental vibrational spectra and the VPT2 spectra calculated on the MP2/def2-TZVPP potential energy surface, therefore, appears to be in part be due to error compensation, underscoring the importance of balancing method and basis set for hydrogen and the transition metal in such calculations.

Table S5. Harmonic vibrational frequencies for $Cu^{+}(H_2O)(H_2)_2$ (wavenumbers in cm^{-1} and assignments) calculated using different method / basis sets combinations.

Mode	PBE0/TZ2P(Slater)	PBE0/TZ2P (Slater) +scalar	MP2/aug-cc-pVTZ	MP2/aug-cc-pVTZ(-PP)	MP2/def2-TZVPP	CCSD(T)/aug-cc-pVTZ(-PP)	CCSD(T)/aug-cc-pVTZ	Mode description
1	126	140	171	167	156	140	108	HOH out-of-plane hindered rotation
2	160	172	179	193	172	149	129	H_2CuH_2 in-plane libration bend
3	207	192	185	199	187	160	142	HOH out-of-plane libration bend
4	248	273	277	314	270	239	242	H_2CuH_2 out-of-plane libration bend
5	288	311	341	379	324	317	281	H2-Cu-H2 bending
6	337	373	410	454	394	347	323	Sym. out-of-plane H_2 hindered rotation
7	340	374	412	467	400	364	326	Antisym. out-of-plane H_2 hindered rotation
8	392	411	425	472	412	419	395	Cu-O stretch
9	569	583	595	615	591	584	565	HOH in-plane libration bend
10	804	859	897	976	880	877	790	Antisym. Cu-H_2 stretch
11	840	912	928	1037	918	1037	821	Sym. Cu-H_2 stretch
12	1185	1276	1366	1493	1310	1309	1178	Antisym. Cu-H_2 stretch

13	1190	1281	1371	1500	1331	1311	1180	Sym. Cu-H_2 stretch
14	1652	1654	1671	1672	1681	1682	1681	H-O-H bend
15	3741	3645	3727	3571	3745	3720	3794	Sym. HH stretch
16	3753	3656	3737	3577	3754	3722	3832	Antisym. HH stretch
17	3817	3814	3780	3778	3809	3793	3835	Sym. OH stretch
18	3899	3895	3877	3874	3905	3877	3878	Antisym. OH stretch

Table S6. H–H distances (pm) and computed H–H stretch frequencies (cm^{-1}) at different levels of theory.

	H–H distance in				
	free H_2	$Cu^+(H_2O)(H_2)$	$Cu^+(H_2O)(H_2)_2$	Δ_1(ads)	Δ_2(ads)
MP2/def2-TZVPP	73.7	79.2	78.9	5.5	5.2
MP2/aug-cc-pVTZ(-PP)	73.7	80.4	78.9	6.7	5.2
CCSD(T)/aug-cc-pVTZ(-PP)	74.3	79.6	79.0	5.3	4.7

Δ_1(ads) and Δ_2(ads): difference between H–H distance in free H_2 and in $Cu^+(H_2O)(H_2)$ and in $Cu^+(H_2O)(H_2)_2$, respectively.

	harmonic H–H stretching frequency in				
	free H_2	$Cu^+(H_2O)(H_2)$	$Cu^+(H_2O)(H_2)_2$	Δ_1(ads)	Δ_2(ads)
MP2/def2-TZVPP	4526	3672	3745<br>3754	-854	-781<br>-772
CCSD(T)/aug-cc-pVTZ(-PP)	4401	3665	3720<br>3722	-736	-681<br>-679

	VPT2 H–H stretching frequency in				
	free H_2	$Cu^+(H_2O)(H_2)$	$Cu^+(H_2O)(H_2)_2$	Δ_1(ads)	Δ_2(ads)
MP2/def2-TZVPP	4298	3481	3565	-817	-733
CCSD(T)/aug-cc-pVTZ(-PP)	4146	–	–	–	–

Δ_1(ads) and Δ_2(ads): difference between the H–H stretching frequencies of free H_2 and H_2 adsorbed in $Cu^+(H_2O)(H_2)$ and $Cu^+(H_2O)(H_2)_2$, respectively.

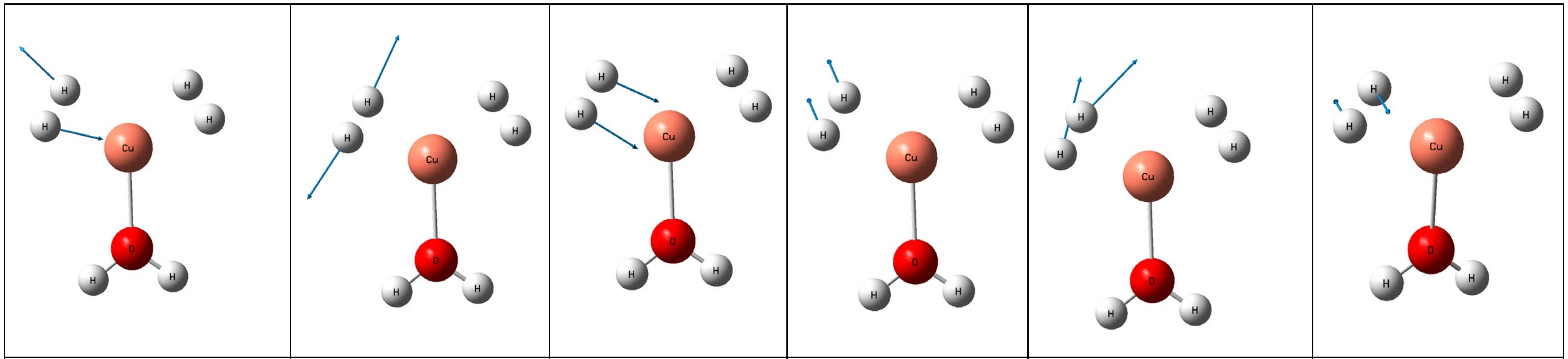

υ_{HCuH} (1307 cm^{-1})	ν_{H_2}(3754 cm^{-1})	ν_{CuH_2}(871 cm^{-1})	$\delta^{IP}_{CuH_2}$(198 cm^{-1})	$\delta^{OOP}_{CuH_2}$(280 cm^{-1})	$\tau^{OOP}_{H_2}$(390 cm^{-1})
υ_{DCuD} (926 cm^{-1})	ν_{D_2}(2659 cm^{-1})	ν_{CuD_2}(617 cm^{-1})	$\delta^{IP}_{CuD_2}$(140 cm^{-1})	$\delta^{OOP}_{CuD_2}$(198 cm^{-1})	$\tau^{OOP}_{D_2}$(276 cm^{-1})
'Hindered translation'	'H-H vibration'	'Cu^+-H_2 vibration'	'Hindered rotation'	'Hindered translation'	'Rotation'

Figure S6: The displacements and corresponding vibrational normal modes of the bound H_2/D_2 to the frozen $Cu^+(H_2O)(H_2)$ fragment. (two vibrations, two hindered translational modes and two hindered rotational mode).

Table S8. Calculated bond dissociation energy (D_e), for the reaction $Cu^+(H_2O)(H_2)_n \rightarrow Cu^+(H_2O)(H_2)_{n-1} + H_2$, ($n$=1,2). All values are in $kJ \cdot mol^{-1}$ and from the bottom of the potential well on the PES, *i.e.* without ZPE correction (Born-Oppenheimer energies).

	$Cu^+(H_2O)(H_2)$	$Cu^+(H_2O)(H_2)_2$
MP2/def2-TZVPP	90.86	28.67
MP2/aug-cc-pVTZ(-PP)	112.29	30.96
MP2/aug-cc-pVQZ(-PP)	113.29	31.13
CCSD(T)/aug-cc-pVTZ(-PP)	96.33	27.71
CCSD(T)/aug-cc-pVQZ(-PP)// CCSD(T)/aug-cc-pVTZ(-PP)	97.23	29.14

Note: The calculated bond dissociation energy (D_e) at the CCSD(T)/aug-cc-pVQZ(-PP) level uses the geometry optimized at the CCSD(T)/ aug-cc-pVTZ(-PP) level.

Table S9. Expectation values <R> (in Å) of bond distance between Cu and the center of the adsorbed hydrogen isotopologues. R_e = 1.6423 Å is the bond length obtained from fitting the data obtained at the CCSD(T)/aug-cc-pVTZ-(PP) level to the Morse.

.

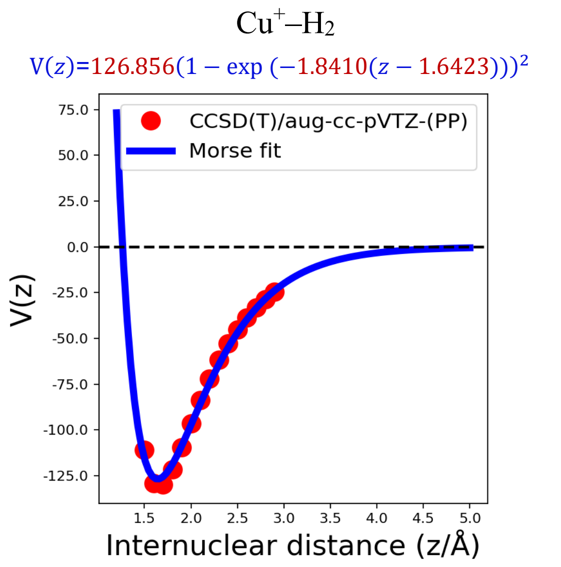

	**<R> (Å)**	**<R>-R_e (Å)**
**H_2**	1.796	0.154
**D_2**	1.768	0.125
**T_2**	1.755	0.113
**HD**	1.779	0.137
**HT**	1.774	0.131
**DT**	1.761	0.119

Table S10. Expectation values <r> (in Å) of bond distance of adsorbed isotopologues. r_e = 0.7904 Å is the bond length obtained after fitting the data obtained at the CCSD(T)/aug-cc-pVTZ-(PP) level to the Morse.

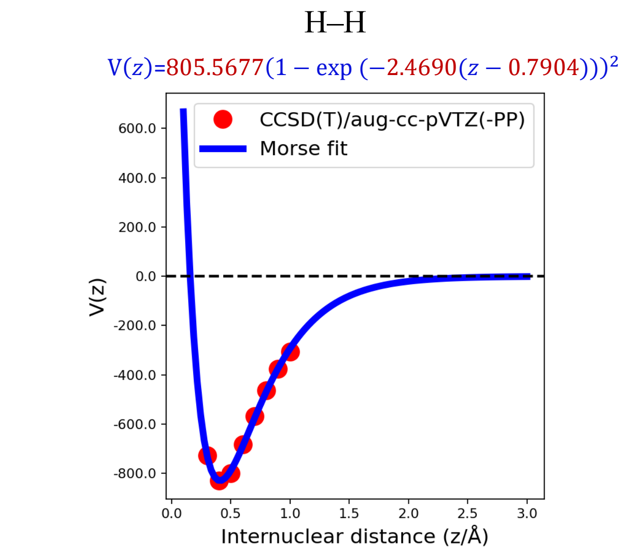

	<r> (Å)	<r>-r_e (Å)
**H_2**	0.8076	0.0172
**D_2**	0.8020	0.0116
**T_2**	0.8009	0.0105
**HD**	0.8043	0.0139
**HT**	0.8035	0.0131
**DT**	0.8014	0.0110

Table S11: Predicted separation factors for dihydrogen isotopologue adsorption at Cu^+, $Cu^+(H_2O)(H_2)$, $Cu^+(H_2O)$ as a function of temperatures.

$Cu^+(H_2O)(H_2)$				
**T(K)**	$\alpha(D_2/H_2)$	$\alpha(HD/H_2)$	$\alpha(DT/D_2)$	$\alpha(T_2/D_2)$
**80**	43.77	8.10	2.06	3.82
**90**	25.64	6.00	1.83	3.06
**100**	16.78	4.74	1.66	2.60
**110**	11.91	3.91	1.54	2.23
**120**	8.98	3.34	1.45	1.99
**130**	7.08	2.93	1.38	1.81
**140**	5.79	2.62	1.32	1.67
**150**	4.88	2.38	1.27	1.56
**160**	4.20	2.19	1.23	1.47
**170**	3.69	2.04	1.20	1.40
**180**	3.29	1.91	1.17	1.34
**200**	2.71	1.72	1.13	1.24
**220**	2.33	1.58	1.09	1.18
**240**	2.05	1.47	1.07	1.12
**260**	1.85	1.39	1.05	1.08

Cu^+			
**T (K)**	$\alpha(D_2/H_2)$	$\alpha(HD/H_2)$	$\alpha(T_2/D_2)$
**80**	17.77	4.35	3.16
**90**	13.80	3.81	2.83
**100**	11.08	3.40	2.57
**110**	9.13	3.07	2.37
**120**	7.70	2.81	2.20
**130**	6.61	2.60	2.06
**140**	5.77	2.42	1.95
**150**	5.10	2.27	1.85
**160**	4.57	2.14	1.76
**170**	4.13	2.03	1.69
**180**	3.76	1.93	1.62
**200**	3.20	1.77	1.52
**220**	2.79	1.65	1.43
**240**	2.48	1.55	1.37
**260**	2.24	1.47	1.31

$Cu^+(H_2O)(H_2)$				
**T (K)**	$\alpha(D_2/H_2)$	$\alpha(HD/H_2)$	$\alpha(DT/D_2)$	$\alpha(T_2/D_2)$
**80**	15.53	4.48	1.62	2.44
**90**	10.35	3.56	1.48	2.06
**100**	7.50	2.97	1.38	1.81
**110**	5.77	2.56	1.30	1.63
**120**	4.64	2.27	1.24	1.49
**130**	3.87	1.04	1.19	1.39
**140**	3.32	1.87	1.15	1.31
**150**	2.91	1.74	1.12	1.24
**160**	2.59	1.63	1.09	1.19
**170**	2.35	1.54	1.07	1.15
**180**	2.15	1.47	1.05	1.11
**200**	1.86	1.35	1.02	1.06
**220**	1.66	1.27	1.00	1.02
**230**	1.58	1.23	0.99	1.00
**240**	1.51	1.20	0.98	0.99
**260**	1.41	1.15	0.97	0.97

$Cu^+(H_2O)$			
**T (K)**	$\alpha(D_2/H_2)$	$\alpha(HD/H_2)$	$\alpha(T_2/D_2)$
**80**	103.39	13.59	5.59
**90**	69.74	10.89	4.74
**100**	49.61	9.00	4.11
**110**	36.85	7.62	3.63
**120**	28.37	6.58	3.26
**130**	22.49	5.78	2.96
**140**	18.29	5.15	2.72
**150**	15.19	4.64	2.52
**160**	12.84	4.23	2.35
**170**	11.03	3.89	2.21
**180**	9.61	4.00	2.08
**200**	7.54	3.15	1.89
**220**	6.14	2.81	1.74
**230**	5.60	2.67	1.67
**240**	5.15	2.55	1.62
**260**	4.42	2.34	1.52

Table S12. Ground state rotational constants A, B and C (in cm^{-1}) of $Cu^+(H_2O)$ and $Cu^+(H_2O)(H_2)_2$ isotopologues.

	harmonic CCSD(T)/aug-cc-pVTZ (-PP)		
	A	B	C
$Cu^+(H_2O)$	14.095	0.297	0.292
$Cu^+(H_2O)(H_2)$	10.889	0.272	0.269
$Cu^+(H_2O)(H_2)_2$	1.796	0.263	0.229
$Cu^+(H_2O)(D_2)_2$	0.961	0.240	0.192
$Cu^+(D_2O)(H_2)_2$	1.591	0.231	0.202
$Cu^+(D_2O)(D_2)_2$	0.899	0.212	0.171

	VPT2 MP2/def2-TZVPP		
	A	B	C
$Cu^+(H_2O)$	13.940	0.299	0.293
$Cu^+(H_2O)(H_2)$	10.978	0.273	0.269
$Cu^+(H_2O)(H_2)_2$	1.818	0.264	0.230
$Cu^+(H_2O)(D_2)_2$	0.976	0.242	0.194
$Cu^+(D_2O)(H_2)_2$	1.610	0.232	0.203
$Cu^+(D_2O)(D_2)_2$	0.912	0.213	0.172

Table S13. H_2 isotopologue desorption energies (kJ·mol^{-1}) for the reaction $Cu^+(H_2O)(H_2)_2 \rightarrow Cu^+(H_2O)(H_2) + H_2$. ZPE corrections from the harmonic approximation.

Species C_{2V} symmetry	**2H₂ and isotopologues attached to $Cu^+(H_2O)$**			
	MP2/def2-TZVPP	MP2/aug-cc-pVTZ (-PP)	MP2/aug-cc-pVQZ (-PP)	CCSD(T)/aug-cc-pVTZ (-PP)
	$\Delta_{des}E(0K)$	$\Delta_{des}E(0K)$	$\Delta_{des}E(0K)$	$\Delta_{des}E(0K)$
$Cu^+(H_2O)(H_2)_2$	17.1	20.2	17.4	14.4
$Cu^+(H_2O)(D_2)_2$	20.3	22.6	21.0	17.6
$Cu^+(H_2O)(T_2)_2$	21.7	23.9	22.7	19.3
$Cu^+(H_2O)(HD)_2$	18.8	21.4	19.4	15.8
$Cu^+(H_2O)(HT)_2$	19.7	22.3	20.4	16.7
$Cu^+(H_2O)(DT)_2$	21.0	23.3	22.0	18.4
**2H₂ and isotopologues attached to $Cu^+(D_2O)$**				
$Cu^+(D_2O)(H_2)_2$	17.3	20.4	17.8	14.3
$Cu^+(D_2O)(D_2)_2$	20.4	22.9	21.5	18.0
$Cu^+(D_2O)(T_2)_2$	21.8	24.3	23.1	19.6
$Cu^+(D_2O)(HD)_2$	19.6	22.0	19.6	16.2
$Cu^+(D_2O)(HT)_2$	20.3	22.7	20.8	17.2
$Cu^+(D_2O)(DT)_2$	21.7	24.0	22.4	18.8
**2H₂ and isotopologues attached to $Cu^+(T_2O)$**				
$Cu^+(T_2O)(H_2)_2$	18.0	20.2	18.0	14.5
$Cu^+(T_2O)(D_2)_2$	20.6	23.1	21.7	18.2
$Cu^+(T_2O)(T_2)_2$	21.9	24.4	23.3	19.9
$Cu^+(T_2O)(HD)_2$	19.7	22.0	20.0	16.4
$Cu^+(T_2O)(HT)_2$	20.5	22.9	20.6	17.4
$Cu^+(T_2O)(DT)_2$	21.8	24.2	22.4	19.0

The ZPEs of free hydrogen isotopologues, namely H_2, D_2, and T_2, are 26.3 kJ·mol^{-1}, 18.6 kJ·mol^{-1}, and 15.2 kJ·mol^{-1}, respectively.

Energy decomposition analysis (EDA) of the Cu^+-H_2 bond

XC-functional benchmark

To determine the appropriate setup for the DFT calculations, we benchmarked the Cu–H_2 bonding energy of complex $Cu(H_2O)(H_2)_2^+$ with different XC functionals against the CCSD(T). We selected a range of XC functionals, including LDA, GGA (BLYP[37], PBE[38]), meta-GGA (TPSS[39],

M06L[40,41]), hybrid (PBE0[42], B3LYP[32]), meta-hybrid (TPSSh[43], M06[40,41], M06-2x[40,41]), range separate hybrid (CAM-B3LYP[44], wB97X-D3[45], LC-BLYP[46]), and double hybrid (PWPB95[47], B2PLYP[48]). Grimme D3[49] dispersion correction was confidently employed for all functionals, and Grimme D4[50] dispersion correction was also confidently utilized for certain functionals. All calculations were performed using the ORCA 4.2.1 program package[51], including the DFT and CCSD(T) reference calculation. The basis set utilized was def2-TZVPP[52], and the RIJCOSX approximation was also employed. Therefore Def2/J was used as auxiliary basis set. The SCF energy convergence criterion was set to 10^{-8} E_H. The benchmark calculations utilized the geometry-optimized structure of H_2, fragment $Cu^+(H_2O)(H_2)$, and complex $Cu^+(H_2O)(H_2)_2$ at the PBE-D3/def2-TZVPP level. The results of the benchmark are presented in **Table S14** and **Figure S7**.

Table S14: Results of the benchmark study for Cu–H_2 bond in $Cu^+(H_2O)(H_2)_2$. All values given in kJ·mol^{-1}.

Functional class	functional	$\Delta E_{bond}(Cu - H_2)$	$\Delta E(DFT) - \Delta E(CCSD(T))$
LDA	LDA-D3	-68.2	-41.5
GGA	PBE-D3	-39.1	-12.4
	PBE-D4	-38.1	-11.4
	BP86-D3	-37.4	-10.7
	BP86-D4	-35.4	-8.7
	BLYP-D3	-28.8	-2.1
	BLYP-D4	-26.9	-0.2
Meta-GGA	TPSS-D3	-34.9	-8.1
	M06L-D3	-37.8	-11.1
Hybrid	B3LYP-D3	-28.2	-1.5
	B3LYP-D4	-26.2	0.5
	PBE0-D3	-34.1	-7.4
Meta-Hybrid	TPSSH-D3	-33.1	-6.4
	M06-D3	-34.8	-8.1
	M06-2x-D3	-24.2	2.6
range separate	CAM-B3LYP-D3	-32.7	-6.0
	wB97X-D3	-33.9	-7.2
	LC-BLYP-D3	-49.2	-22.5
double-hybrid	PWPB95-D3	-26.2	0.5
	B2PLYP-D3	-26.5	0.3
Reference	CCSD(T)	-26.7	

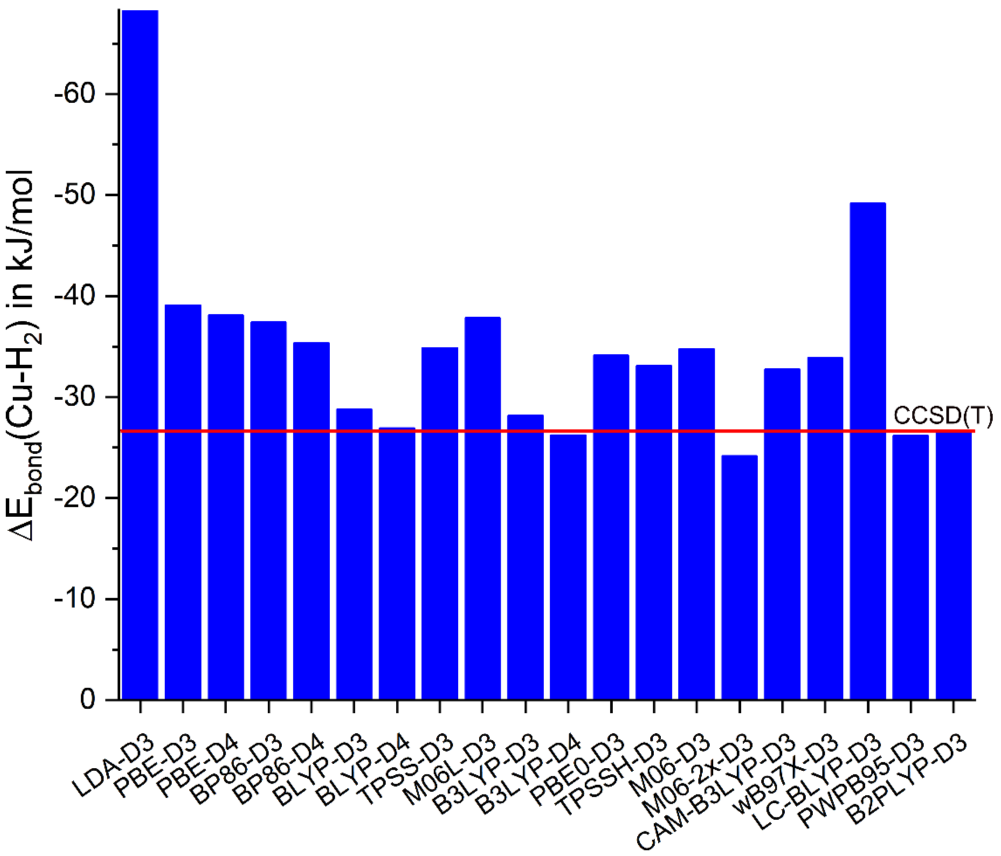

Figure S7: Bonding energy of Cu-H_2 bond in complex $Cu^{+}(H_2O)(H_2)_2$ in kJ/mol. The red line corresponds to CCSD(T) reference calculation.

The CCSD(T) results demonstrate deviations of less than 1 kJ·mol^{-1} for the double hybrid functional, BLYP, and B3LYP with D4 dispersion correlation. Due to a missing implementation, double hybrid functionals cannot be utilized for Energy Decomposition Analysis (EDA) calculations. Therefore, B3LYP-D4 was selected for bond analysis.

Stuctures

For the EDA-NOCV (Energy Decomposition Analysis with Natural Orbital for Chemical Valence extension), all systems ($Cu(H_2)_4^{+}$, $Cu(H_2)_2(H_2O)^{+}$, $Cu(H_2)(H_2O)^{+}$, $Cu(H_2)^{+}$ and $Cu(H_2O)^{+}$) and fragments were optimized. The optimized structures of the studied complexes are shown in the **Figure S8**.

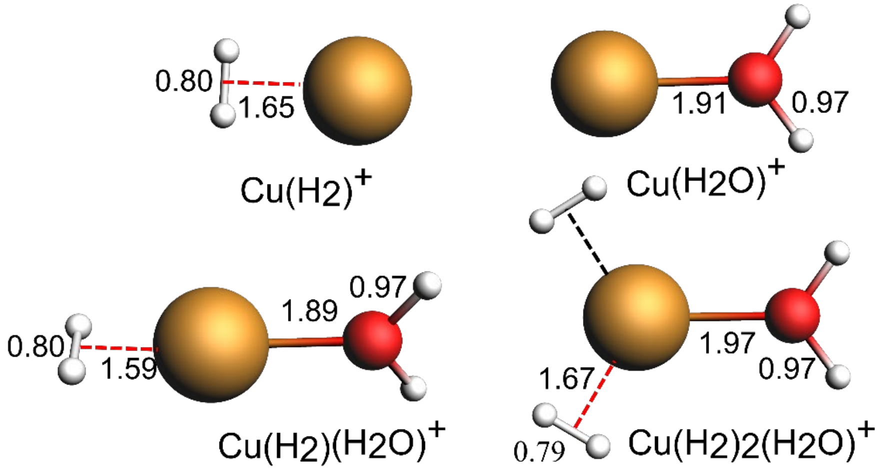

Figure S8: The optimized structure of the studied complexes, whereby all Cu–H_2O and Cu–H_2 bonds (red) are analyzed by EDA. All bond lengths are in Å.

Energy decomposition analysis with natural orbital for chemical valence (EDA-NOCV)

The settings for the EDA calculation are described in the methods section of the article. The EDA results are shown in **Table S15**.

Table S15: EDA result for Cu-H_2O bond of $(Cu(H_2)_2(H_2O)^+$, $Cu(H_2)(H_2O)^+$ and $Cu(H_2O)^+$ and the Cu-O distance r(Cu-H_2O).[a]

	$Cu(H_2)_2(H_2O)^+$		$Cu(H_2)(H_2O)^+$		$Cu(H_2O)^+$	
ΔE_{int}	-162		-208		-190	
ΔE_{int}(disp)[b]	-6	4 %	-4	2 %	-4	2 %
ΔE_{int}(elec)[b]	-156	96 %	-204	98 %	-186	98 %
ΔE_{elstat}[c]	-226	68 %	-268	67 %	-273	66 %
ΔE_{Pauli}	178		196		225	
ΔE_{orb}[c]	-107	32 %	-131	33 %	-137	34 %
ΔE_1[d]	-62	58 %	-71	54 %	-73	53 %
ΔE_2[d]	-16	15 %	-21	16 %	-29	21 %

ΔE_3[d]	-12	11 %	-16	12 %	-16	12 %
ΔE_3[d]	-12	11 %	-16	12 %	-15	11 %
ΔE_{prep}	25		2		1	
$\Delta E_{prep}(H_2O)$	1		1		1	
$\Delta E_{prep}(Cu^+L_{0\text{-}3})$	24		1		0	
ΔE_{bond}	-137		-206		-189	
$r(Cu - H_2O)$	1.97		1.90		1.91	

[a] All energies in kJ/mol, bond distances in Å computed with B3LYP-D4/TZ2P. Fragments are closed-shell species $Cu^+L_{0\text{-}3}$ and H_2 (L=H_2O, H_2) in Table S15 and $Cu^+L_{0\text{-}2}$ and H_2O (L= H_2) in Table S16. [b] Percentage values give the relative contributions of dispersion and electronic effects to ΔE_{int}. [c] Percentage values give the relative contributions to the attractive pEDA terms ΔE_{elstat} and ΔE_{orb}. [d] Sum over all NOCV contributions corresponds to ΔE_{orb}.

Bonding analysis of the Cu^+–H_2O bond also shows a dative character for all $Cu(H_2O)(H_2)_n^+$ (n = 0, 1, 2), in which the dispersion interaction makes only a small contribution (about -5 kJ·mol^{-1}). The Cu^+–H_2O bond is about 100 kJ·mol^{-1} stronger than the Cu^+–H_2 bond. Only complex $Cu(H_2O)(H_2)_2^+$ has a significant value for preparation energy due to the configuration change in the Cu fragment. Additionally the Cu^+–O bond distance for $Cu(H_2O)(H_2)_2^+$ is significantly larger than for the other two complexes correspond to a weaker interaction. For $Cu(H_2O)(H_2)^+$ and $Cu(H_2O)^+$, the Cu–O bond length is similar (1.90 Å and 1.91 Å, respectively). However, the interaction energy (and binding energy) is about 20 kJ·mol^{-1} higher for $Cu(H_2)(H_2O)^+$ due to the significantly lower Pauli repulsion (30 kJ·mol^{-1}) for this complex, which explains the back bonding to the H_2 ligand. This reduces the electron density in the HOMO and HOMO-1, resulting in less repulsion with occupied orbitals of water. Orbital and electrostatic interactions are slightly stronger for $Cu(H_2O)^+$ (5 kJ·mol^{-1} each). The weaker electrostatic and orbital interactions for $Cu(H_2)(H_2O)^+$ can be explained by the reduction of the charge at the Cu center due to the sigma donation of the LUMO by H_2 ligands.

Figure S9 shows the four most important NOCVs for the Cu–H_2O bonds. More than 90% of the ΔE_{orb} is accounted for by these NOCVs. The NOCVs for all investigated Cu–H_2O bonds have the same shape but differ in their contributions to the ΔE_{orb} and the amount of charge transfer. All NOCV results indicate that there is a transfer of charge from the O atom in the water molecule to the Cu atom. Specifically, NOCV 1 represents the σ interaction between the free electron pair on

the O atom in the bonding direction and the empty s orbital on the Cu, while NOCV 2 represents the corresponding p orbital in the bonding direction of the Cu-atom. NOCV 4 shows the π interaction of the other free electron pair with the p orbital of the Cu atom. Finally, NOCV 3 describes the π interaction between the binding p orbital of the O atom in water and the last p orbital on the Cu.

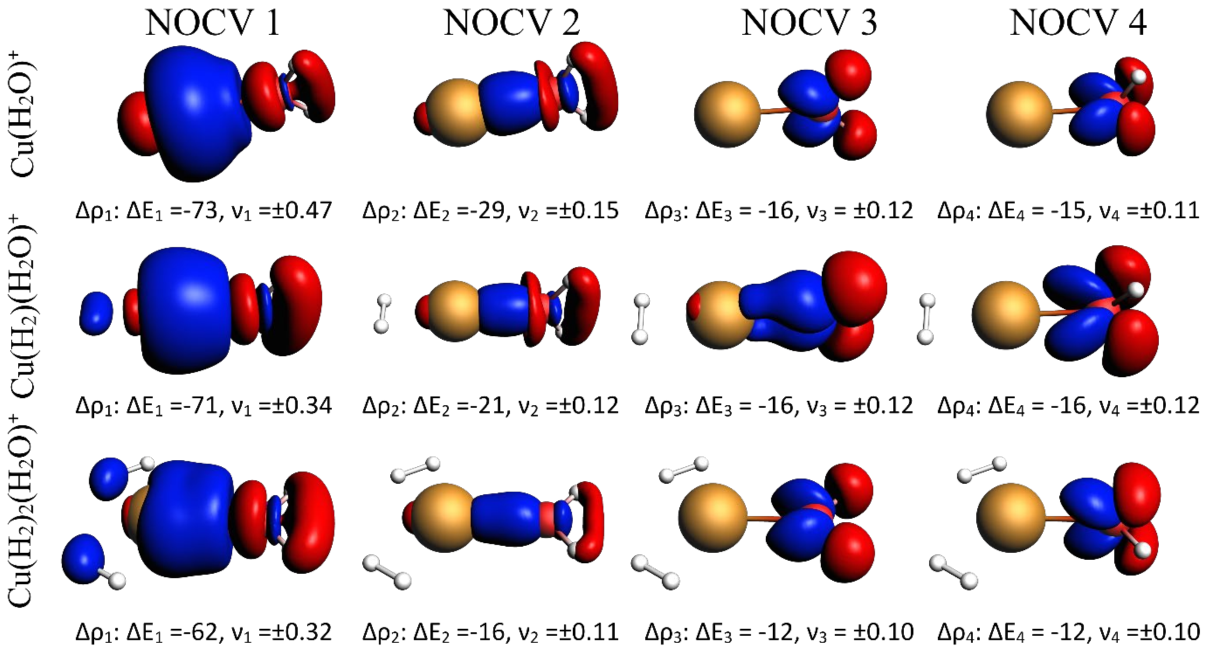

Figure S11: Selected deformation densities ($\Delta\rho_i$) from EDA-NOCVs for Cu-H_2O bond with energy contribution (ΔE_i) to ΔE_{orb} in kJ/mol and eigenvalues (ν_i). Charge depletion (red) and charge accumulation (blue) with isosurface value 0.0020 for $Cu(H_2O)^+$, 0.0011 for $Cu(H_2)(H_2O)^+$ and 0.0015 for $Cu(H_2)_2(H_2O)^+$.

1 a) D. Sengupta, P. Melix, S. Bose, J. Duncan, X. Wang, M. R. Mian, K. O. Kirlikovali, F. Joodaki, T. Islamoglu, T. Yildirim, R. Q. Snurr and O. K. Farha, *J. Am. Chem. Soc.*, 2023, **145**, 20492; b) B. R. Barnett, H. A. Evans, G. M. Su, H. Z. H. Jiang, R. Chakraborty, D. Banyeretse, T. J. Hartman, M. B. Martinez, B. A. Trump, J. D. Tarver and others, *J. Am. Chem. Soc.*, 2021, **143**, 14884;

2 R. Xiong, L. Zhang, P. Li, W. Luo, T. Tang, B. Ao, G. Sang, C. Chen, X. Yan, J. Chen and M. Hirscher, *Chem. Eng. J.*, 2020, **391**, 123485.

3 B. Ipek, R. A. Pollock, C. M. Brown, D. Uner and R. F. Lobo, *J. Phys. Chem. C*, 2018, **122**, 540.

4 I. Weinrauch, I. Savchenko, D. Denysenko, S. M. Souliou, H.-H. Kim, M. Le Tacon, L. L. Daemen, Y. Cheng, A. Mavrandonakis, A. J. Ramirez-Cuesta, D. Volkmer, G. Schütz, M. Hirscher and T. Heine, *Nat. Commun.*, 2017, **8**, 14496.

5 J. Teufel, H. Oh, M. Hirscher, M. Wahiduzzaman, L. Zhechkov, A. Kuc, T. Heine, D. Denysenko and D. Volkmer, *Adv. Mater.*, 2013, **25**, 635.

6 H. Oh and M. Hirscher, *Eur. J. Inorg. Chem.*, 2016, **2016**, 4278.

7 T. Wulf and T. Heine, *Int. J. Quatum. Chem.*, 2018, **118**, e25545.

8 T. Wulf and T. Heine, *J. Phys. Chem. C*, 2020, **124**, 9409.

9 a) K. R. Asmis, A. Fielicke, G. von Helden and G. Meijer, in *Atomic clusters*, ed. D. Woodruff, Elsevier, Amsterdam and Boston, 2007, pp. 327–375; b) E. J. Bieske and O. Dopfer, *Chem. Rev.*, 2000, **100**, 3963; c) J. M. Lisy, *Int. Rev. Phys. Chem.*, 1997, **16**, 267; d) M. A. Duncan, *Int. Rev. Phys. Chem.*, 2003, **22**, 407; e) N. Heine and K. R. Asmis, *Int. Rev. Phys. Chem.*, 2015, **34**, 1;

10 a) G. Piccini and J. Sauer, *J. Chem. Theory. Comput.*, 2014, **10**, 2479; b) K. Sillar, A. Hofmann and J. Sauer, *J. Am. Chem. Soc.*, 2009, **131**, 4143;

11 a) F. Dahlmann, C. Lochmann, A. N. Marimuthu, M. Lara-Moreno, T. Stoecklin, P. Halvick, M. Raoult, O. Dulieu, R. Wild, S. Schlemmer and others, *J. Chem.Phys.*, 2021, **155**; b) F. Dahlmann, P. Jusko, M. Lara-Moreno, P. Halvick, A. N. Marimuthu, T. Michaelsen, R. Wild, K. Geistlinger, S. Schlemmer, T. Stoecklin, R. Wester and S. Brünken, *Mol. Phys.*, 2022, **120**;

12 J. Jin, T. Wulf, M. Jorewitz, T. Heine and K. R. Asmis, *Phys. Chem. Chem. Phys.*, 2023, **25**, 5262.

13 P. R. Kemper, P. Weis, M. T. Bowers and P. Ma\^\itre, *J. Am. Chem. Soc.*, 1998, **120**, 13494.

14 P. Maitre and C. W. Bauschlicher, *J. Phys. Chem.*, 1993, **97**, 11912.

15 a) R. H. Crabtree, *Chem. Rev.*, 2016, **116**, 8750; b) G. J. Kubas, *Metal dihydrogen and s-bond complexes: structure, theory, and reactivity*, Springer Science & Business Media, 2001;

16 N. Heine and K. R. Asmis, *Int. Rev. Phys. Chem.*, 2015, **34**, 1.

17 K. R. Asmis, M. Brümmer, C. Kaposta, G. Santambrogio, G. von Helden, G. Meijer, K. Rademann and L. Wöste, *Phys. Chem. Chem. Phys.*, 2002, **4**, 1101.

18 a) T. B. Ward, E. Miliordos, P. D. Carnegie, S. S. Xantheas and M. A. Duncan, *J. Chem.Phys.*, 2017, **146**, 224305; b) J. H. Marks, E. Miliordos and M. A. Duncan, *J. Chem.Phys.*, 2021, **154**, 64306; c) P. D. Carnegie, B. Bandyopadhyay and M. A. Duncan, *J. Chem.Phys.*, 2011, **134**;

19 P. D. Carnegie, McCoy, AB and M. A. Duncan, *J. Phys. Chem. A*, 2009, **113**, 4849.

20 T. Ziegler and A. Rauk, *Theoret. Chim. Acta*, 1977, **46**, 1.

21 K. Kitaura and K. Morokuma, *Int. J. Quatum. Chem.*, 1976, **10**, 325.

22 F. M. Bickelhaupt and E. J. Baerends, in *Reviews in Computational Chemistry*, ed. K. B. Lipkowitz and D. B. Boyd, Wiley, 2000, vol. 15, pp. 1–86.

23 L. Zhao, M. von Hopffgarten, D. M. Andrada and G. Frenking, *WIREs Comput. Mol. Sci.*, 2018, **8**.

24 M. P. Mitoraj, A. Michalak and T. Ziegler, *J. Chem. Theory. Comput.*, 2009, **5**, 962.

25 P. M. Morse, *Phys. Rev.*, 1929, **34**, 57.

26 M. Mayer and K. R. Asmis, *J. Phys. Chem. A*, 2021, **125**, 2801.

27 N. Heine and K. R. Asmis, *Int. Rev. Phys. Chem.*, 2016, **35**, 507.

28 J. F. Stanton, J. Gauss, L. Cheng, M. E. Harding, D. A. Matthews, and P. G. Szalay, Cfour.

29 M. J. Frisch, G. W. Trucks, H. B. Schlegel, G. E. Scuseria, M. A. Robb, J. R. Cheeseman, G. Scalmani, V. Barone, G. A. Petersson, H. Nakatsuji, X. Li, M. Caricato, A. Marenich, J. Bloino, B. G. Janesko, R. Gomperts, B. Mennucci, H. P. Hratchian, J. V. Ortiz, A. F. Izmaylov, J. L. Sonnenberg, D. W. Young, F. Ding, F. Lipparini, F. Egidi, J. Goings, B. Peng, A. Petrone, T. Henderson, D. Ranasinghe, V. G. Zakrzewski, J. Gao, N. Rega, G. Zheng, W. Liang, M. Hada, M. Ehara, K. Toyota, R. Fukuda, J. Hasegawa, M. Ishida, T. Nakajima, Y. Honda, O. Kitao, H. Nakai, T. Vreven, K. Throssell, J. A. Montgomery, J. E. Peralta, F. Ogliaro, M. Bearpark, J. J. Heyd, E. Brothers, K. N. Kudin, V. N. Staroverov, T. Keith, R. Kobayashi, J. Normand, K. Raghavachari, A. Rendell, J. C. Burant, S. S. Iyengar, J. Tomasi, M. Cossi, J. M. Millam, M. Klene, C. Adamo, R. Cammi, J. W. Ochterski, R. L. Martin, K. Morokuma, O.

Farkas, J. B. Foresman and D. J. Fox, Gaussian 16 (Revision C.01), Gaussian Inc., Wallingford CT, 2016.

30 T. Ziegler and A. Rauk, *Theoret. Chim. Acta*, 1977, **46**, 1.

31 G. te Velde, F. M. Bickelhaupt, E. J. Baerends, C. Fonseca Guerra, S. J. A. van Gisbergen, J. G. Snijders and T. Ziegler, *J. Comput. Chem.*, 2001, **22**, 931.

32 P. J. Stephens, F. J. Devlin, C. F. Chabalowski and M. J. Frisch, *The Journal of Physical Chemistry*, 1994, **98**, 11623.

33 E. Caldeweyher, S. Ehlert, A. Hansen, H. Neugebauer, S. Spicher, C. Bannwarth and S. Grimme, *The Journal of Chemical Physics*, 2019, **150**, 154122.

34 E. van Lenthe and E. J. Baerends, *J. Comput. Chem.*, 2003, **24**, 1142.

35 E. van Lenthe, A. Ehlers and E.-J. Baerends, *The Journal of Chemical Physics*, 1999, **110**, 8943.

36 a) A. D. Becke, *Phys. Rev. A*, 1988, **38**, 3098; b) J. P. Perdew, *Phys. Rev. B*, 1986, **33**, 8822;

37 J. P. Perdew, K. Burke and M. Ernzerhof, *Phys. Rev. Lett.*, 1996, **77**, 3865.

38 V. N. Staroverov, G. E. Scuseria, J. Tao and J. P. Perdew, *The Journal of Chemical Physics*, 2003, **119**, 12129.

39 Y. Zhao and D. G. Truhlar, *The Journal of Chemical Physics*, 2006, **125**, 194101.

40 Y. Zhao and D. G. Truhlar, *Theoret. Chim. Acta*, 2008, **120**, 215.

41 M. Ernzerhof and G. E. Scuseria, *The Journal of Chemical Physics*, 1999, **110**, 5029.

42 J. P. Perdew, J. Tao, V. N. Staroverov and G. E. Scuseria, *The Journal of Chemical Physics*, 2004, **120**, 6898.

43 T. Yanai, D. P. Tew and N. C. Handy, *Chemical Physics Letters*, 2004, **393**, 51.

44 Y.-S. Lin, G.-D. Li, S.-P. Mao and J.-D. Chai, *J. Chem. Theory. Comput.*, 2013, **9**, 263.

45 Y. Tawada, T. Tsuneda, S. Yanagisawa, T. Yanai and K. Hirao, *The Journal of Chemical Physics*, 2004, **120**, 8425.

46 L. Goerigk and S. Grimme, *J. Chem. Theory. Comput.*, 2011, **7**, 291.

47 S. Grimme, *The Journal of Chemical Physics*, 2006, **124**, 34108.

48 S. Grimme, J. Antony, S. Ehrlich and H. Krieg, *The Journal of Chemical Physics*, 2010, **132**, 154104.

49 E. Caldeweyher, J.-M. Mewes, S. Ehlert and S. Grimme, *Phys. Chem. Chem. Phys.*, 2020, **22**, 8499.

50 F. Neese, F. Wennmohs, U. Becker and C. Riplinger, *The Journal of Chemical Physics*, 2020, **152**, 224108.

51 F. Weigend and R. Ahlrichs, *Physical Chemistry Chemical Physics*, 2005, **7**, 3297.

52 a) E. J. Baerends, D. E. Ellis and P. Ros, *Chemical Physics*, 1973, **2**, 41; b) B. I. Dunlap, J. W. D. Connolly and J. R. Sabin, *The Journal of Chemical Physics*, 2008, **71**, 3396; c) K. Eichkorn, O. Treutler, H. Öhm, M. Häser and R. Ahlrichs, *Chemical Physics Letters*, 1995, **242**, 652; d) C. van Alsenoy, *J. Comput. Chem.*, 1988, **9**, 620;
